# Tissue-Specific Effects of Reduced β-catenin Expression on *Adenomatous Polyposis Coli* Mutation-Instigated Tumorigenesis in Mouse Colon and Ovarian Epithelium

**DOI:** 10.1371/journal.pgen.1005638

**Published:** 2015-11-03

**Authors:** Ying Feng, Naoya Sakamoto, Rong Wu, Jie-yu Liu, Alexandra Wiese, Maranne E. Green, Megan Green, Aytekin Akyol, Badal C. Roy, Yali Zhai, Kathleen R. Cho, Eric R. Fearon

**Affiliations:** 1 Department of Internal Medicine, University of Michigan Medical School, Ann Arbor, Michigan, United States of America; 2 Department of Pathology, University of Michigan Medical School, Ann Arbor, Michigan, United States of America; 3 Department of Human Genetics, University of Michigan Medical School, Ann Arbor, Michigan, United States of America; Erasmus University Medical Center, NETHERLANDS

## Abstract

*Adenomatous polyposis coli* (*APC*) inactivating mutations are present in most human colorectal cancers and some other cancers. The APC protein regulates the β-catenin protein pool that functions as a co-activator of T cell factor (TCF)-regulated transcription in Wnt pathway signaling. We studied effects of reduced dosage of the *Ctnnb1* gene encoding β-catenin in *Apc*-mutation-induced colon and ovarian mouse tumorigenesis and cell culture models. Concurrent somatic inactivation of one *Ctnnb1* allele, dramatically inhibited *Apc* mutation-induced colon polyposis and greatly extended *Apc*-mutant mouse survival. *Ctnnb1* hemizygous dose markedly inhibited increases in β-catenin levels in the cytoplasm and nucleus following *Apc* inactivation in colon epithelium, with attenuated expression of key β-catenin/TCF-regulated target genes, including those encoding the EphB2/B3 receptors, the stem cell marker Lgr5, and Myc, leading to maintenance of crypt compartmentalization and restriction of stem and proliferating cells to the crypt base. A critical threshold for β-catenin levels in TCF-regulated transcription was uncovered for *Apc* mutation-induced effects in colon epithelium, along with evidence of a feed-forward role for β-catenin in *Ctnnb1* gene expression and *CTNNB1* transcription. The active β-catenin protein pool was highly sensitive to *CTNNB1* transcript levels in colon cancer cells. In mouse ovarian endometrioid adenocarcinomas (OEAs) arising from *Apc*- and *Pten*-inactivation, while *Ctnnb1* hemizygous dose affected β-catenin levels and some β-catenin/TCF target genes, *Myc* induction was retained and OEAs arose in a fashion akin to that seen with intact *Ctnnb1* gene dose. Our findings indicate *Ctnnb1* gene dose exerts tissue-specific differences in *Apc* mutation-instigated tumorigenesis. Differential expression of selected β-catenin/TCF-regulated genes, such as *Myc*, likely underlies context-dependent effects of *Ctnnb1* gene dosage in tumorigenesis.

## Author Summary

Enhanced Wnt signaling contributes to colorectal and other cancers. β-catenin functions in Wnt signaling as a T cell factor (TCF) transcriptional co-activator. Previous studies showed specific β-catenin dosage favors Wnt signaling-dependent tumorigenesis for some tumor types. However, earlier studies emphasized the role of constitutional *Ctnnb1* and *Apc* gene variations, rather than somatic gene targeting, and the work focused on small intestine tumors and no effects on colon tumor phenotypes were described. Furthermore, definitive insights were lacking into how reduced *Ctnnb1* gene dosage affected *Apc* mutation-dependent tumorigenesis. Here, we show somatic inactivation of one *Ctnnb1* allele dramatically inhibits mouse colon adenomatous polyposis induced by somatic bi-allelic *Apc* inactivation. In contrast, *Ctnnb1* hemizygous inactivation does not affect mouse ovarian endometrioid adenocarcinoma development arising from *Apc*- and *Pten*-inactivation. *Ctnnb1* hemizygous gene dose dramatically reduces the active pool of β-catenin, leading to the significant inhibition of β-catenin/TCF-regulated target gene expression, including those encoding key stem cell regulatory and crypt compartmentalization factors in colon epithelium. Tissue-specific differences for expression of selected β-catenin/TCF-regulated genes, such as *Myc*, may contribute to the context-dependent effects of *Ctnnb1* gene dosage in *Apc* mutation-driven colon and ovarian tumors.

## Introduction

Colorectal cancers (CRCs) harbor accumulated mutations in tumor suppressor genes and oncogenes along with epigenetic alterations. Many CRCs arise from precursor lesions, such as adenomatous polyps or serrated epithelial lesions with dysplasia. Inactivating mutations in the *APC* (adenomatous polyposis coli) and *TP53* tumor suppressor genes are found in roughly 80% and 60% of CRCs, respectively [1]. Oncogenic mutations activating the functions of the KRAS and PI3KCA (phosphoinositide-3-kinase, catalytic, alpha polypeptide) proteins are found in about 40% and 20% of CRCs, respectively [1]. Constitutional mutations inactivating one *APC* allele underlie the familial adenomatous polyposis (FAP) syndrome, where affected individuals often develop hundreds to thousands of colon adenomas during their second or third decades of life. The wild type *APC* allele is somatically inactivated in adenomas arising in those with FAP [1, 2]. Mice carrying certain heterozygous, constitutional mutations inactivating *Apc*, such as the *Apc* ^*Min*^ mutation, may develop 50–100 small intestinal tumors and occasional colon tumors by 140 days of age and nearly all of the tumors are adenomas. Similar to the situation in FAP tumors, intestinal tumors in *Apc* ^*Min*^ mice show somatic inactivation of the wild type *Apc* allele [3].

The best understood function of the roughly 300 kD APC protein is regulation of the pool of β-catenin protein that functions in the canonical (β-catenin-dependent) Wnt signaling pathway [4–6]. In the absence of an activating Wnt ligand signal, the β-catenin destruction complex—comprised by the APC, AXIN, casein kinase I, and glycogen synthase kinase-*3*β factors and other proteins—promotes phosphorylation of conserved serine/threonine residues in the β-catenin amino (N)-terminal region. The N-terminally phosphorylated β-catenin can then be β ubiquitinated and degraded by the proteasome. Activating Wnt ligands inhibit degradation of the “free” or Wnt signaling pool of β-catenin via binding at the cell surface to the frizzled and LRP5/6 (low density lipoprotein-related proteins 5 and 6) cognate receptor complex, resulting in inhibition of β-catenin phosphorylation and/or ubiquitination by the destruction complex [4, 6]. In colon adenomas and CRCs where both *APC* alíeles are defective, destruction of the free pool of β-catenin is impaired and active β-catenin accumulates in the cytoplasm and nucleus, where it can complex with DNA binding proteins of the TCF (T-cell factor family)/Lef (lymphoid enhancer family) family. β-catenin functions as a transcriptional co-activator for TCFs [7]. Normally, β-catenin/TCF transcriptional activation is restricted to the crypt base, especially in the so-called crypt base columnar stem cells characterized by expression of the Wnt-regulated Lgr5 presumptive stem cell marker protein [8]. Constitutive activation of β-catenin/TCF transcription in Wnt pathway-defective adenomas and CRCs may promote a stem or progenitor cell phenotype in epithelial cells independent of cell position in the crypt [9, 10]. Activation of β-catenin/TCF-dependent transcription also alters crypt compartmentalization and coordinated migration of cells, apparently through increased expression of the EphB2 and EphB3 receptors and via inhibition of the expression of their ligands ephrin B1 and B2 [11, 12]. The *MYC* gene has been highlighted as a potentially key target gene regulated by β-catenin/TCF in CRCs. Genes encoding negative-feedback inhibitor proteins functioning in the Wnt/β-catenin/TCF pathway, such as AXIN2, DKK1, and NKD1, are also activated by β-catenin/TCF (see http://www.stanford.edu/~rnusse/pathways/targets.html for a list of candidates). In *APC*-mutant neoplastic cells, the ability of these induced regulator proteins to inhibit the Wnt signaling pathway is abrogated because the factors function upstream of or at the level of the APC protein in the pathway [13].

Besides these findings, other evidence indicates that *APC* inactivation may promote cancer development through β-catenin dysregulation. For instance, while most CRCs harbor *APC* mutations, a subset of CRCs and other cancers lacking *APC* mutations have *CTNNB1* gene mutations resulting in production of oncogenic β-catenin proteins that are resistant to regulation by the destruction complex and that activate β-catenin/TCF transcription [6, 13]. Also, some prior studies have used genetic approaches to study effects of *Ctnnb1* gene dosage on liver, small intestine, and mammary gland tumor phenotypes in mouse models as well as effects of *Ctnnb1* hemizygous inactivation state (*Ctnnb1* ^*+/-*^) in *Apc*-mutation induced mouse embryonic development phenotypes [14, 15]. The prior studies indicated the *Ctnnb1* ^*+/-*^ constitutional state can inhibit intestinal and liver tumorigenesis in mice carrying mutations in the *Apc* gene (*Apc* ^*1638N*^, *Apc* ^*Min*^, or *Apc* ^*fl*^) [14, 15]. In contrast, mammary gland tumorigenesis was enhanced in *Apc* ^*1638N*^ *Ctnnb1* ^*+/-*^ mice, perhaps because *Ctnnb1* functions as a tumor suppressor gene in the mammary gland tumors via β-catenin’s role in E-cadherin-dependent tumor suppression [14]. Nonetheless, while the prior studies yielded evidence that β-catenin signaling dosage impacts *Apc* mutation-induced tumorigenesis in some tissues, the prior work did not assess the role of *Ctnnb1* dosage in *Apc* mutation-induced colon tumorigenesis, the chief site of *APC* mutation-dependent tumorigenesis in humans. Moreover, the work used mice constitutionally deficient in β-catenin, not just in *Apc*-mutant epithelial cells, and the findings did not highlight specific factors and mechanisms that might account for effects of *Ctnnb1* dosage in *Apc* mutation-instigated tumorigenesis in different contexts. We report here on studies of the effects of *Ctnnb1* gene dosage on β-catenin protein expression and β-catenin/TCF transcription in *Apc* mutation-induced colon and ovarian mouse tumors and cell culture models. We provide evidence that *Apc* mutation-induced tumorigenesis in the colon is inhibited by *Ctnnb1* hemizygous gene status through marked effects on the free pool of β-catenin in the cytoplasm and nucleus and its ability to activate key β-catenin/TCF-regulated target genes, including those encoding key stem factors, such as Lgr5, and regulators of crypt compartmentalization, such as the EphB2/B3 receptors. We also uncovered a novel feed-forward mechanism where β-catenin protein stabilization and β-catenin/TCF transcription appear critical in regulating *Ctnnb1/CTNNB1* transcription in the setting of *Apc* inactivation in mouse colon and human colon cancer cells. Moreover, we found that differences in the ability to activate *Myc* expression may underlie colon versus ovary tissue-specific differences in *Apc* mutation-instigated tumorigenesis in the setting of *Ctnnb1* hemizygous gene dosage.

## Results

### Inactivation of a *Ctnnb1* allele extends survival and inhibits adenomatous polyposis and epithelial abnormalities induced by somatic bi-allelic *Apc* inactivation in mouse colon

**Fig 1:**
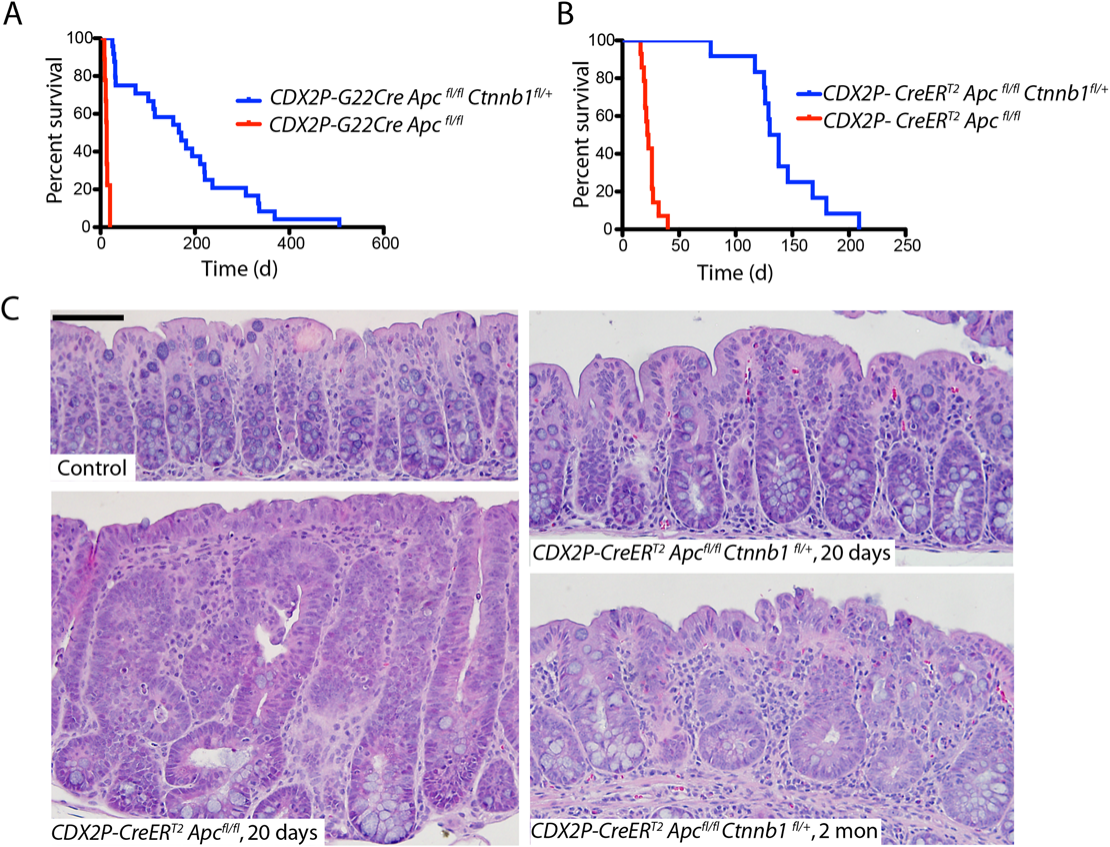
*Apc* mutation-induced polyposis in mouse cecum and colon epithelium is dramatically inhibited by concurrent inactivation of one *Ctnnb1* allele. (A) The survival of *CDX2P-G22Cre Apc* ^*fl/fl*^ *Ctnnb1* ^*fl/+*^ mice (blue; *n* = 24) is contrasted with that *of CDX2P-G22Cre Apc* ^*fl/fl*^ mice (red; *n* = 9). *P <0*.*0001* by log-rank (Mantel-Cox) test. (B) The survival of *CDX2P-CreER* ^*T2*^ *Apc* ^*fl/fl*^ *Ctnnb1* ^*fl/+*^ mice (blue; *n* = 12) is contrasted with that in *CDX2P-CreER* ^*T2*^ *Apc* ^*fl/fl*^ mice (red; *n* = 14), where time zero represents the third day of three days when TAM was administered daily. *P <0*.*0001* by log-rank (Mantel-Cox) test. (C) Hematoxylin and eosin (H&E) staining of proximal colon tissues from a *CDX2P-CreER* ^*T2*^ *Apc* ^*fl/fl*^ mouse 20 days after the third of three daily doses of TAM (left, lower), and a control (non-gene-targeted) mouse (left, upper). Right panels show H&E staining of proximal colon tissues from *CDX2P-CreER* ^*T2*^ *Apc* ^*fl/fl*^ *Ctnnb1* ^*fl/+*^ mice 20 days (right, upper) or 2 months (right, lower) after TAM dosing. Scale bars, 50 μm.

We previously described *CDX2P-G22Cre* transgenic mice, in which human *CDX2* regulatory sequences and an out-of-frame *Cre* transgene allele, carrying a 22-basepair guanine nucleotide repeat tract affecting the Cre open reading frame, manifest mosaic Cre recombinase expression in caudal embryonic tissues and in epithelium of the distal ileum, cecum, colon, and rectum during adult life [16]. We also previously described *CDX2P-CreER* ^*T2*^ transgenic mice that express a tamoxifen (TAM)-regulated Cre protein (*CreER* ^*T2*^) under control of human *CDX2* regulatory sequences, allowing for TAM-inducible targeting of loxP-containing alleles in adult terminal ileum, cecum, colon, and rectal epithelium [17]. Using the *CDX2P-G22Cre* or *CDX2P-CreER* ^*T2*^ transgenic mice, we have described the phenotypic consequences in colon epithelium of somatic, bi-allelic, inactivating mutations in *Apc* [16, 17]. Consistent with our prior studies, we found *CDX2P-G22Cre Apc* ^*fl/fl*^ mice lived only for 8–20 days after birth (median survival = 13 d; Fig 1A). After three daily doses of TAM to inactivate both *Apc* alleles in distal intestinal epithelial tissues, *CDX2P-CreER* ^*T2*^ *Apc* ^*fl/fl*^ adult mice lived on average for 22 days (Fig 1B). In marked contrast, concurrent somatic inactivation of one *Ctnnb1* allele along with both *Apc* alleles, using either the *CDX2P-G22Cre or CDX2P-CreER* ^*T2*^ transgene for somatic gene targeting, led to a dramatically increased life span relative to that seen in mice with *Apc* bi-allelic targeting, with median survival of 168 d of age in *CDX2P-G22Cre Apc* ^*fl/fl*^ *Ctnnb1* ^*fl/+*^ mice and for 134 d after TAM treatment in the *CDX2P-CreER* ^*T2*^ *Apc* ^*fl/fl*^ *Ctnnb1* ^*fl/+*^ mice (Fig 1A and 1B).

Consistent with our prior reports [16, 17], the proximal colon and cecum of both *CDX2P-G22Cre Apc* ^*fl/fl*^ mice (when moribund at 8–20 d of age) and *CDX2P-CreER* ^*T2*^ *Apc* ^*fl/fl*^ mice (only 20 days after TAM induction) were dramatically thickened and many polypoid lesions were seen (S1 Fig). Histological analysis of proximal colon epithelial tissues from these mice showed significant hyperplastic and dysplastic (adenomatous) changes along with frequent crypt fission and branching (Fig 1C). The dramatic polyposis in cecum and colon seen following bi-allelic *Apc* inactivation was significantly inhibited at both early and later time points by concurrent inactivation of one *Ctnnb1* allele, with no grossly discernable epithelial phenotype seen in the proximal colon and only two to four polyps in the cecum per mouse as the *CDX2P-G22Cre Apc* ^*fl/fl*^ *Ctnnb1* ^*fl/+*^ and *CDX2P-CreER* ^*T2*^ *Apc* ^*fl/fl*^ *Ctnnb1* ^*fl/+*^ mice were aged (S1 Fig). The cecal polyps arising in *CDX2P-G22Cre Apc* ^*fl/fl*^ *Ctnnb1* ^*fl/+*^ and *CDX2P-CreER* ^*T2*^ *Apc* ^*fl/fl*^ *Ctnnb1* ^*fl/+*^ mice may contribute to their premature mortality relative to control mice, as no other grossly detectable intestinal lesions or pathology were noted in the mice. The Cre-mediated somatic inactivation of both *Apc* alleles and one *Ctnnb1* allele in proximal colon epithelium was confirmed by genotyping. The rare cecal adenomas arising in *CDX2P-CreER* ^*T2*^ *Apc* ^*fl/fl*^ *Ctnnb1* ^*fl/+*^ mice were found to have significant fractions of cells that escaped Cre-mediated *Ctnnb1* targeting, even though Cre-mediated somatic inactivation of both *Apc* alleles occurred to the same extent in the rare adenomas and proximal colon mucosa (S1 Fig). Compared to the situation in *CDX2P-CreER* ^*T2*^ *Apc* ^*fl/fl*^ mice, microscopic examination of proximal colon tissues of *CDX2P-CreER* ^*T2*^ *Apc* ^*fl/fl*^ *Ctnnb1* ^*fl/+*^ mice revealed modest hyperplastic changes and minimal crypt branching (Fig 1C). Our efforts to inactivate both *Ctnnb1* alleles in colon epithelium via either *CDX2P-G22Cre*- or *CDXP-CreER* ^*T2*^-mediated targeting with or without *Apc* inactivation indicated that colon epithelial cells completely lacking β-catenin expression and function could not be generated. This likely reflects a required role for β-catenin function in colon epithelium, perhaps not limited to Wnt signaling, but also in cadherin-mediated adhesion, centrosome assembly or other functions.

**Fig 2:**
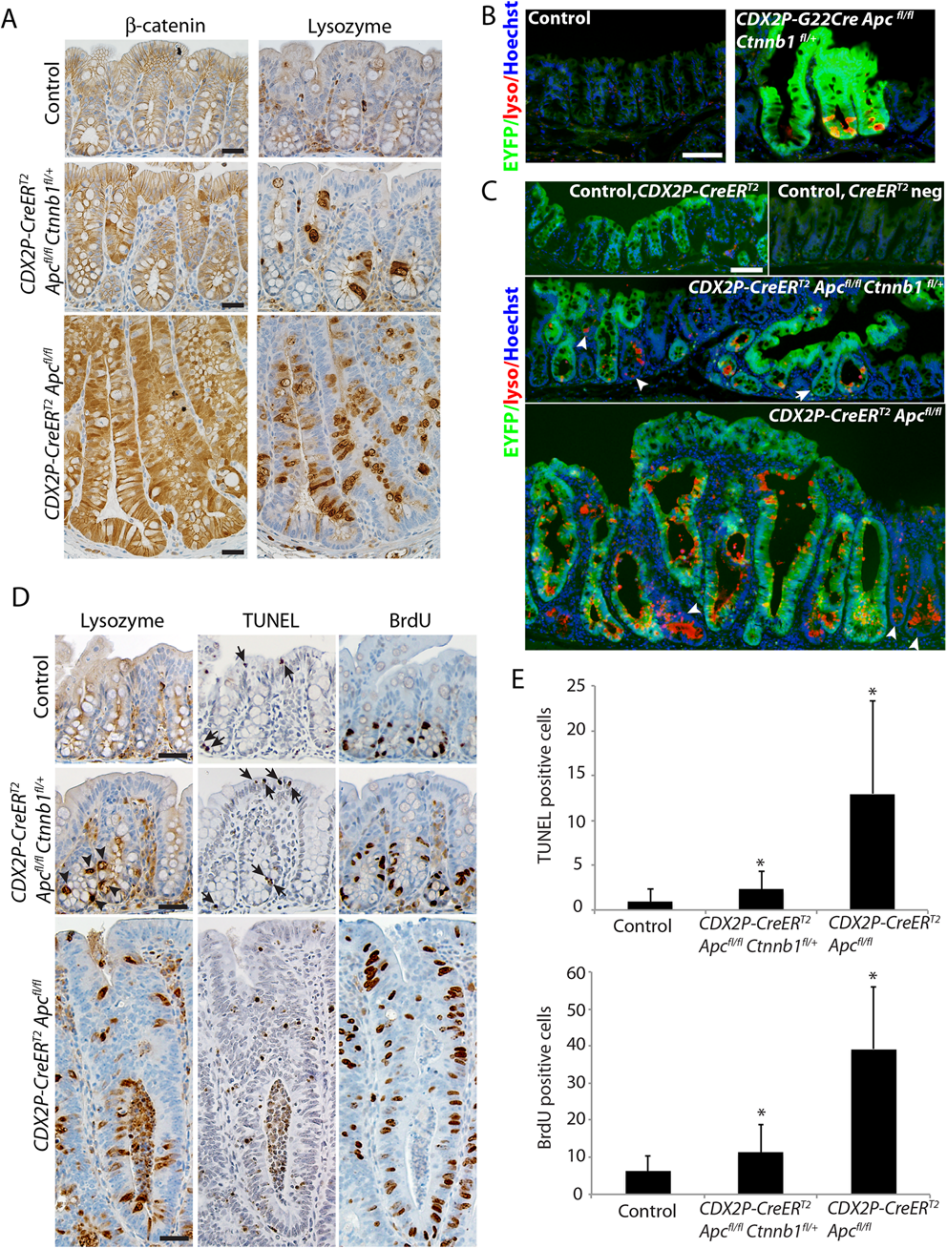
Inactivation of a *Ctnnb1* allele in mouse colon epithelium reduces apoptosis and hyperproliferation induced by bi-allelic *Apc* inactivation, and also reduces, but does not abrogate, the aberrant cell differentiation associated with *Apc* inactivation. (A) Representative immunohistochemical staining for β-catenin (left panels) and lysozyme (right panels) in a *CDX2P-CreER* ^*T2*^ *Apc* ^*fl/fl*^ *Ctnnb1* ^*fl/+*^ mouse (middle row) or a *Cdx2P-CreER* ^*T2*^ *Apc* ^*fl/fl*^ mouse (bottom row) 20 days following three daily doses of TAM. Immunohistochemical staining is also shown for normal control colon mucosa from a *Cre* negative control littermate mouse (top row). Scale bars, 20 μm. (B and C) EYFP expression activated in targeted colon crypts by Cre-mediated recombination was imaged along with Immunofluorescence staining for lysozyme (lyso; red) in proximal colon tissues that were counter-stained with Hoechst 33342 for detection of nuclei. A 6-month old *G22Cre Apc* ^*fl/fl*^ *Ctnnb1* ^*fl/+*^ *EYFP* ^*fl/+*^ mouse and a *Cre* negative control littermate are shown in panel B. Colon tissues from a *CDX2P-CreER* ^*T2*^ *Apc* ^*fl/fl*^ *Ctnnb1* ^*fl/+*^ *EYFP* ^*fl/+*^ mouse (panel C, middle) or a *CDX2P-CreER* ^*T2*^ *Apc* ^*fl/fl*^ *EYFP* ^*fl/+*^ mouse (panel C, bottom) were analyzed 38 days after the third of three daily doses of TAM. Colon tissues from a *CDX2P-CreER* ^*T2*^ *EYFP* ^*fl/+*^ mouse (panel C, top left) and a *Cre*-negative *EYFP* reporter mouse (panel C, top right) served as controls. Arrowheads indicate the EYFP-negative crypts that expressed the Paneth cell maker, lysozyme. An arrow in the middle panel indicates a crypt showing EYFP expression but no lysozyme staining. Scale bars, 50 μm. (D) TUNEL assay (middle column panels) and immunohistochemical staining for lysozyme (left column panels) and BrdU (right column panels) in proximal colon tissues of a *CDX2P-CreER* ^*T2*^ *Apc* ^*fl/fl*^ *Ctnnb1* ^*fl/+*^ mouse (middle row) or a *Cdx2P-CreER* ^*T2*^ *Apc* ^*fl/fl*^ mouse (bottom row) 20 days after three daily doses of TAM. The normal colon mucosa from a cre negative mouse served as control (top row). Arrows indicate apoptotic cells in tissues from a *CDX2P-CreER* ^*T2*^ *Apc* ^*fl/fl*^ *Ctnnb1* ^*fl/+*^ mouse and the control mouse; arrowheads indicate the lysozyme-positive cells in tissues from a *CDX2P-CreER* ^*T2*^ *Apc* ^*fl/fl*^ *Ctnnb1* ^*fl/+*^ mouse. Scale bars, 25 μm. (E) Apoptotic cells per crypt in proximal colon, based on the TUNEL assay, were quantified (panel *E*, top; n = 3 and >100 crypts counted; *P < .001 compared to the control mouse). Proliferating cells per crypt, as assessed by BrdU incorporation, was quantified (panel *E*, bottom; n = 3 and >110 crypts counted; *P < .001 compared to the control mouse).

Immunohistological analysis of colon sections from *CDX2P-CreER* ^*T2*^ *Apc* ^*fl/fl*^ mice showed strong cytoplasmic and nuclear β-catenin expression in many epithelial cells, compared to the nearly uniform membrane β-catenin staining in colon epithelium of wild-type mice (Fig 2A). In *CDX2P-CreER* ^*T2*^ *Apc* ^*fl/fl*^ *Ctnnb1* ^*fl/+*^ mice, we observed infrequent cells with elevated cytoplasmic and/or nuclear β-catenin expression (Fig 2A). Paneth cells, a specialized secretory cell linage that expresses lysozyme and other markers, are found at the crypt base in normal mouse small intestinal epithelium, but are absent in normal mouse colon epithelium. Paneth cells have been proposed to have a key role in generation and/or maintenance of the intestinal crypt stem cell niche [18]. Bi-allelic *Apc* inactivation has been associated with the generation of many ectopic lysozyme-expressing Paneth-like cells throughout the crypts of small intestine and colon [12, 17, 19, 20]. We confirmed this finding in *CDX2P-CreER* ^*T2*^ *Apc* ^*fl/fl*^ mice (Fig 2A). Whereas no Paneth-like cells were seen in normal mouse colon, modest numbers of lysozyme-expressing cells were seen in the colons of *CDX2P-CreER* ^*T2*^ *Apc* ^*fl/fl*^ *Ctnnb1* ^*fl/+*^ mice (Fig 2A). We also used a transgenic mouse line carrying a Cre-activated enhanced yellow fluorescence protein (EYFP) reporter gene at the ubiquitously expressed *Rosa26* locus to monitor colon epithelial cells and glands where Cre-mediated targeting had occurred. Ectopic lysozyme-expressing cells were found in nearly all of the EYFP-positive crypts in *CDX2P-G22Cre Apc* ^*fl/fl*^ *Ctnnb1* ^*fl/+*^ and *CDX2P-CreER* ^*T2*^ *Apc* ^*fl/fl*^ *Ctnnb1* ^*fl/+*^ mice (Fig 2B and 2C). The rare occurrence of Paneth-like cells in crypts without EYFP expression likely reflects the possibility that Cre may more efficiently target the loxP sites at the *Apc* locus than at the *Rosa26* locus.

Prior studies from other groups and ours have shown that *Apc* bi-allelic inactivation increases both cell proliferation and apoptosis in intestine and colon epithelium [12, 17, 19, 21]. Following TAM-induced *Apc* bi-allelic inactivation in proximal colon epithelium, we confirmed significantly elongated crypts and increased cell proliferation and apoptosis relative to control epithelial tissues (Fig 2D and 2E). In contrast, only modestly increased crypt height, cell proliferation and apoptosis relative to control epithelium were seen in epithelium of *CDX2P-CreER* ^*T2*^ *Apc* ^*fl/fl*^ *Ctnnb1* ^*fl/+*^ mice following combined *Apc* and *Ctnnb1* gene inactivation (Fig 2D and 2E). Furthermore, although bi-allelic *Apc* inactivation induced extensive cell proliferation in the upper half of targeted colon crypts, cell proliferation following gene targeting in *CDX2P-CreER* ^*T2*^ *Apc* ^*fl/fl*^ *Ctnnb1* ^*fl/+*^ mice was largely restricted to the bottom half of each crypt, with only a slight increase in cell proliferation compared to control colon epithelium (Fig 2D and 2E). The cell proliferation and apoptosis results were well correlated with the immunohistochemical studies of β-catenin levels and localization (Fig 2A), suggesting differences in the strength of β-catenin-dependent Wnt signaling in cells with bi-allelic *Apc* defects underlie the observed effects on colon epithelial morphology, cell fate and differentiation, and cell proliferation and apoptosis.

The orientation of the mitotic spindle axis may impact on cell fate decisions in intestinal epithelium. At cytokinesis, the orientation of the spindle axis in a planar fashion (i.e., parallel to the crypt axis) is thought to generate two daughter cells with equivalent luminal (apical) and basement (extracellular matrix) surfaces. If the spindle axis is not oriented parallel to the crypt axis, cytokinesis generates daughter cells with differences in luminal and basement membrane surfaces and the potential for resultant differences in the fates adopted by the two daughter cells. We previously reported significant increases in the percentage of epithelial cells where the mitotic spindle axis was oriented orthogonal to the planar axis in *Apc*-mutant mouse colon crypts relative to wild type crypts [17]. Consistent with our prior results, in colon epithelium of *CDX2P-CreER* ^*T2*^ *Apc* ^*fl/fl*^ mice treated with TAM to inactivate both *Apc* alleles, roughly 50% of the cells in mitosis had mitotic spindle axes ≥30° degrees out of the planar axis, with nearly 20% showing spindle axes between 60° and 90° out of planar alignment. In contrast, in epithelium of *CDX2P-CreER* ^*T2*^ *Apc* ^*fl/fl*^ *Ctnnb1* ^*fl/+*^ mice and control mice, >75% of mitotic colon epithelial cells had their mitotic spindles aligned within 30° of the planar (crypt) axis (S2 Fig). The findings indicate β-catenin levels have a key role in the altered mitotic spindle axis phenotype of *Apc*-mutant colon epithelium.

### *Ctnnb1* inactivation inhibits *Apc* mutation-induced colon tumorigenesis via maintenance of EphB/ephrinB signaling and restriction of presumptive stem cells to the crypt base

**Fig 3:**
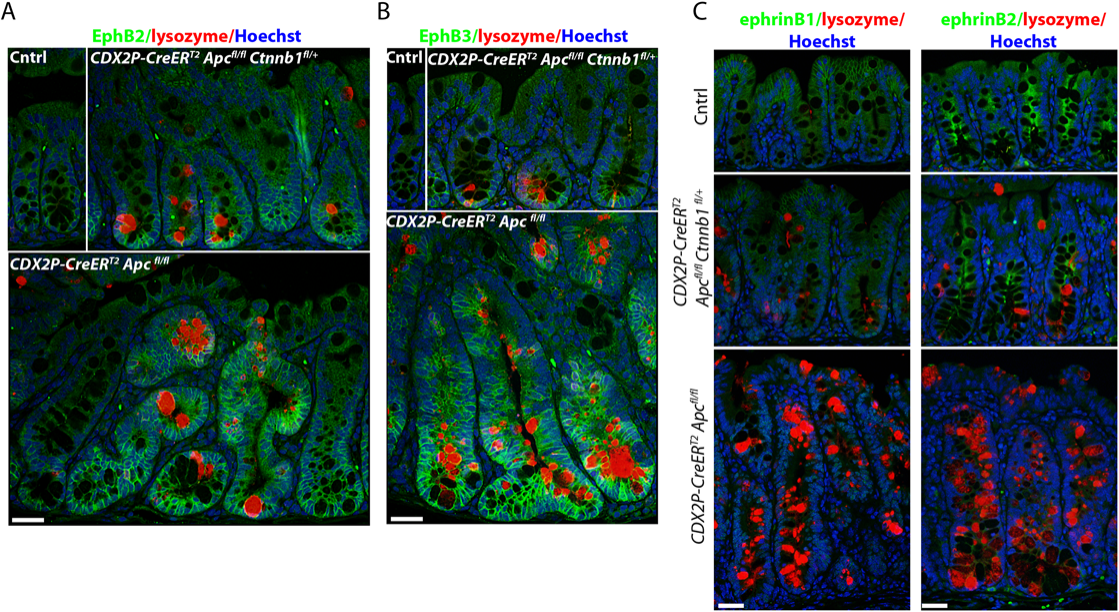
Inactivation of one *Ctnnb1* allele in *Apc*-mutant mouse colon epithelium preserves the compartmentalization of the EphB2/B3- and ephrinB1/B2-expressing cells to maintain crypt architecture. Mouse proximal colon tissue sections were stained for lysozyme (red) and EphB2 (panel A, green) or EphB3 (panel B, green) receptors, and counter-stained with Hoechst 33342 (Blue) to identify nuclei. The tissues from a *CDX2P-CreER* ^*T2*^ *Apc* ^*fl/fl*^ *Ctnnb1* ^*fl/+*^ mouse (top, right) and a *CDX2P-CreER* ^*T2*^ *Apc* ^*fl/fl*^ mouse (bottom) were analyzed 20 days after the third of three daily doses of TAM. The normal colon mucosa from a *Cre*-negative littermate was used as a control (Cntrl, top left). Scale bars, 20 μm. (C) Proximal colon tissue sections from the same mice were co-stained for ephrinB1 (left, green) or ephrinB2 (right, green) and lysozyme (red), and counter-stained with Hoechst 33342 (Blue). Scale bars, 20 μm.

As described above, bi-allelic *Apc* inactivation acutely induces hyperproliferation and dysplastic alterations in mouse proximal colon epithelium, with the altered epithelium arising in part from expansion of the crypt progenitor compartment at the expense of the differentiated compartment, along with frequent crypt fission/branching [12, 17, 21]. The EphB/ephrinB signaling axis has been implicated in control of intestinal epithelial cell compartmentalization along the crypt axis and in cell migration [11, 22]. The EphB2 and EphB3 receptors are two key effectors of compartmentalization and cell migration in the crypt, and EphB2 and EphB3 are each encoded by a gene activated in intestinal tissues by β-catenin/Tcf transcription. The EphB2/B3 receptor ligands, ephrinB1 and ephrinB2, show highest expression levels in differentiated cells at the crypt surface, and expression of ephrins B1 and B2 is negatively regulated by β-catenin/Tcf activity [11, 23]. Of note, EphB-ephrinB interactions generate repulsive forces that separate and compartmentalize the EphB- and ephrinB-expressing cells to maintain crypt architecture [11, 23]. In normal mouse colon epithelium, the EphB2 and EphB3 receptors were expressed only in progenitor cells at the crypt base (Fig 3A and 3B). We found bi-allelic *Apc* inactivation in colon epithelium not only increased EphB2 and EphB3 expression, but also perturbed the gradient of EphB2 and B3 receptor expression along the crypt axis, with EphB2/B3 expression seen even at the crypt surface in *Apc*-mutant crypts (Fig 3A and 3B). In the case of ephrin ligand expression, our studies demonstrated strong expression of ephrinB1 and B2 in normal colon surface epithelial cells and normal colon crypt cells other than the crypt base. The normal pattern of ephrinB1/B2 expression remained largely unaffected in *Apc*-mutant crypts with one *Ctnnb1* allele inactivated (Fig 3C). In contrast, in *Apc*-mutant crypts where *Ctnnb1* dosage was intact, ephrinB1/B2 expression was markedly down-regulated in colon surface epithelial cells and throughout the crypt (Fig 3C). Our findings are consistent with those in a prior study that showed increased expression of EphB2/B3 and loss of ephrinB1/B2 expression in colon adenomas of *Apc* ^*min/+*^ mice [23]. Although expression of EphB2/B3 was moderately elevated in some colon epithelial cells of *CDX2P-CreER* ^*T2*^ *Apc* ^*fl/fl*^ *Ctnnb1* ^*fl/+*^ mice compared to control mice, elevated EphB2/B3 expression remained restricted to the crypt base region, rather than spreading throughout the crypt as was seen in Apc-mutant crypts with intact *Ctnnb1* gene dosage (Fig 3A and 3B). This observation suggests the reduced β-catenin levels in *CDX2P-CreER* ^*T2*^ *Apc* ^*fl/fl*^ *Ctnnb1* ^*fl/+*^ mice leads to a failure to induce enough β-catenin/TCF-regulated EphB2/B3 expression to overcome the repulsive effects of the retained expression of ephrinB1/B2 ligands in *Apc*-mutant crypts with reduced *Ctnnb1* dosage.

A similar expression pattern to that seen for EphB2 and EphB3 was also found for Sox9, a transcription factor encoded by a β-catenin/Tcf target gene. Sox9 expression is restricted to stem/progenitor cells at the normal colon crypt base (S3 Fig). Sox9 expression was only modestly increased and expanded following gene targeting in crypts of *CDX2P-CreER* ^*T2*^ *Apc* ^*fl/fl*^ *Ctnnb1* ^*fl/+*^ mice relative to the marked changes in Sox9 levels and the number of Sox9-expressing cells in crypts from *CDX2P-CreER* ^*T2*^ *Apc* ^*fl/fl*^ mice (S3 Fig). In spite of the reduced increase in β-catenin levels in colon crypts of *CDX2P-CreER* ^*T2*^ *Apc* ^*fl/fl*^ *Ctnnb1* ^*fl/+*^ mice relative to *CDX2P-CreER* ^*T2*^ *Apc* ^*fl/fl*^ mice, the resultant signaling was still sufficient to generate some ectopic Paneth-like cells (Fig 3A and 3B). In addition, the modest increase in β-catenin levels in targeted crypts of *CDX2P-CreER* ^*T2*^ *Apc* ^*fl/fl*^ *Ctnnb1* ^*fl/+*^ mice was sufficient to induce expression in targeted crypts of a β-galactosidase reporter gene integrated into the β-catenin/TCF-regulated *Axin2* locus. However, β-galactosidase expression was reduced in crypts and few if any colon surface epithelial cells expressed β-galactosidase in *Apc*-mutant epithelium with hemizgyous *Ctnnb1* dosage (S3 Fig). In contrast, uniformly strong β-galactosidase expression was seen throughout *Apc*-mutant colon crypts and surface epithelial cells with intact *Ctnnb1* dosage (S3 Fig). Taken together, the findings indicate distinct β-catenin/Tcf target genes in colon epithelium display differing transcriptional responses to β-catenin levels, with *Axin2* perhaps representing a target gene capable of being activated by modest to moderate levels of β-catenin in colon epithelium. The *Sox9*, *EphB2*, *and EphB3* genes appear dependent on higher levels of β-catenin for transcriptional activation in colon epithelium.

**Fig 4:**
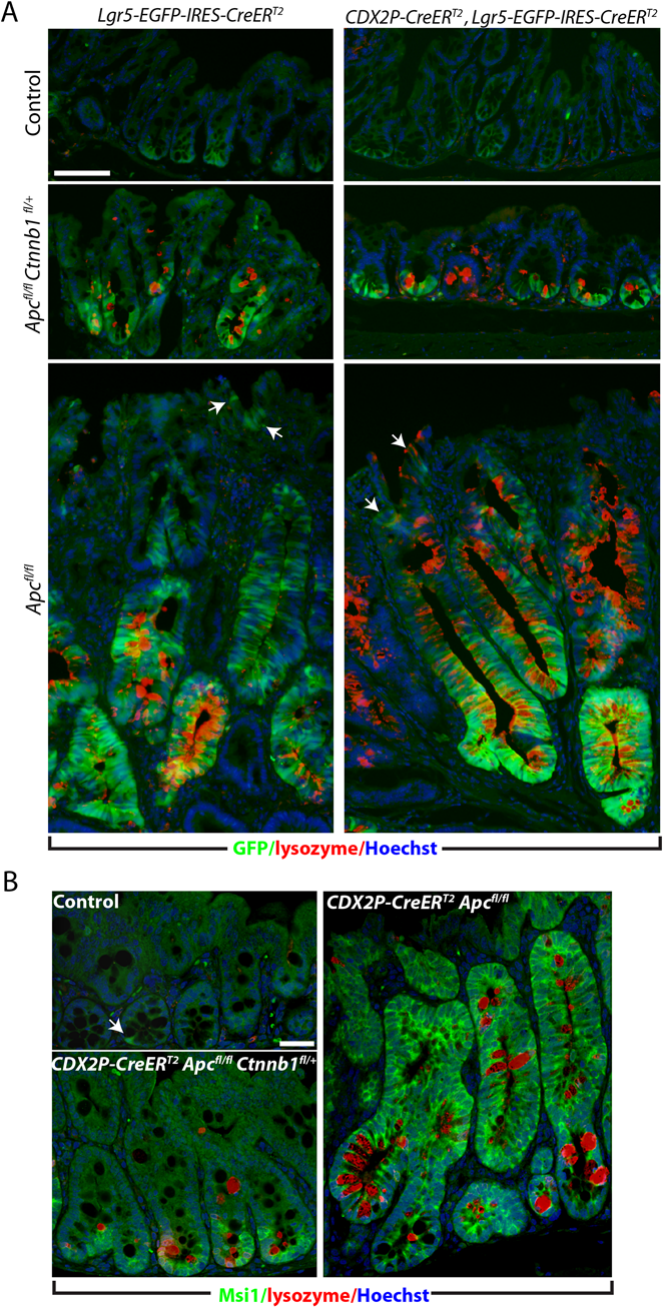
Inactivation of a *Ctnnb1* allele in *Apc*-mutant mouse colon epithelium reduces aberrant expansion of the presumptive stem cell pool expressing Msi1 or Lgr5. (A) GFP imaging (green) plus Immunofluorescence staining for lysozyme (red) in proximal colon tissues, counter-stained with Hoechst 33342 (blue). Tissues from *Lgr5-EGFP-IRES-CreER* ^*T2*^ *Apc* ^*fl/fl*^ mice (bottom row of panels), *Lgr5-EGFP-IRES-CreER* ^*T2*^ *Apc* ^*fl/fl*^ *Ctnnb1* ^*fl/+*^ mice (middle row of panels), or control *Lgr5-EGFP-IRES-CreER* ^*T2*^ mice (top row of panels), carrying (right columns) or not carrying (left columns) the *CDX2P-CreER* ^*T2*^ transgene, were analyzed after 20 days TAM dosing. Arrows indicate the cells positive for Lgr5 at regions close to the luminal surface in colon tissues from a mouse with *Apc* inactivation. Scale bar, 50 μm. (B) Mouse proximal colon tissues were co-stained for Msi1 (green) and lysozyme (red), and counter-stained with Hoechst 33342 (Blue). The *CDX2P-CreER* ^*T2*^ *Apc* ^*fl/fl*^ *Ctnnb1* ^*fl/+*^ mouse (left, bottom) and the *CDX2P-CreER* ^*T2*^ *Apc* ^*fl/fl*^ mouse (right) were analyzed 20 days after TAM dosing. The normal colon mucosa from a *Cre*-negative mouse was used as a control (left, top). The arrow indicates a rare Msi1-positive cell near the crypt base in normal colon epithelium, whereas Msi1-positive cells are more evident in the *Apc*-mutant crypts. Scale bars, 20 μm.

To address mechanisms underlying suppression of crypt fission and branching in *Apc*-deficient colon epithelium when one *Ctnnb1* allele was inactivated, we compared expression of presumptive stem cell markers in *Apc*-deficient colon crypts where both *Ctnnb1* alleles were intact or where only one *Ctnnb1* allele was active. Consistent with our prior work [17], 20 days after TAM-induced bi-allelic *Apc* inactivation, we detected strong induction of enhanced green fluorescent protein (EGFP) expressed from the *Lgr5* locus (*Lgr5-EGFP*) (Fig 4A) in *Apc*-deficient colon epithelium generated by *CDX2P-CreER* ^*T2*^ targeting. *Lgr5* is a β-catenin/TCF-regulated gene and a marker of presumptive crypt base columnar stem cells in normal colon, and the *Lgr5* allele that we used has a EGFP open reading frame integrated in the locus to allow for monitoring of endogenous Lgr5 expression [8]. In *Apc*-mutant epithelium, we also confirmed strong induction of the Msi1 RNA-binding protein (Fig 4B), another presumptive intestinal stem cell marker [24, 25]. In contrast to a prior study where it was reported that Lgr5-expressing cells were only expanded at the lower part of the crypts in colon epithelium following mutant β-catenin induction [21], we detected EYFP-positive and Msi-positive cells essentially throughout the *Apc*-mutant dysplastic colon crypts when both *Ctnnb1* alleles were active, though expression of EYFP was more prominent near the crypt base region, including in the *de novo* crypts. While the net number of EYFP- and Msi1-expressing cells per crypt were slightly increased (e.g. from 3–4 to 5–8 Lgr5-positive cells per crypt) in colon epithelium of TAM-treated *CDX2P-CreER* ^*T2*^ *Apc* ^*fl/fl*^ *Ctnnb1* ^*fl/+*^ mice compared to the control mice, paralleling the subtle increase in crypt fission/budding seen, the expanded population of EYFP-positive cells remained restricted to the crypt base region (Fig 4A), consistent with the EphB2 and EphB3 data described above. EYFP expression patterns in colon similar to those seen in TAM-treated *CDX2P-CreER* ^*T2*^ *Apc* ^*fl/fl*^ *Ctnnb1* ^*fl/+*^ mice were also obtained when we used TAM-treatment to activate the *Lgr5-*driven *CreER* ^*T2*^ transgene to target *Apc* and *Ctnnb1* alleles and EYFP expression was used to mark Lgr5-expressing cells (Fig 4A).

Consistent with our studies of Lgr5 and Msi expression patterns in colon epithelium, the levels of transcripts encoding presumptive stem cell markers, including Lgr5, CD44, Msi1, and Hopx, were also found to increase dramatically in the colon tissues of *CDX2P-CreER* ^*T2*^ *Apc* ^*fl/fl*^ mice (S4 Fig). The induction of genes encoding stem cell markers and other selective β-catenin/Tcf target genes (such as *Axin2*, *Nkd1*, *Ccnd1* and *Irs1*) observed in *Apc*-deficient colon epithelium was significantly suppressed in colon epithelium from *CDX2P-CreER* ^*T2*^ *Apc* ^*fl/fl*^ *Ctnnb1* ^*fl/+*^ mice (S4 Fig). Taken together, our data indicate that the robust induction of many β-catenin/Tcf-regulated genes that is seen response to *Apc* inactivation was variably inhibited in reduced *Ctnnb1* gene dosage and β-catenin protein levels in mouse colon epithelium. In the setting of inactivation of one *Ctnnb1* allele, the inability of *Apc* inactivation to substantially activate certain key β-catenin/TCF-regulated genes with functions in colon crypt compartmentalization and cell migration (e.g., *EphB2* and *EphB3*) or stem cell fate (e.g., *Lgr5* and *Msi)* is likely to underlie the dramatic abrogation of adenoma formation in *CDX2P-CreER* ^*T2*^ *Apc* ^*fl/fl*^ *Ctnnb1* ^*fl/+*^ mice.

### *Ctnnb1* gene dosage effects on *Myc* and *Ctnnb1* gene expression in Apc-mutant mouse colon tissues and an apparent feed-forward mechanism for β-catenin/TCF in regulating *Ctnnb1* transcription

**Fig 5:**
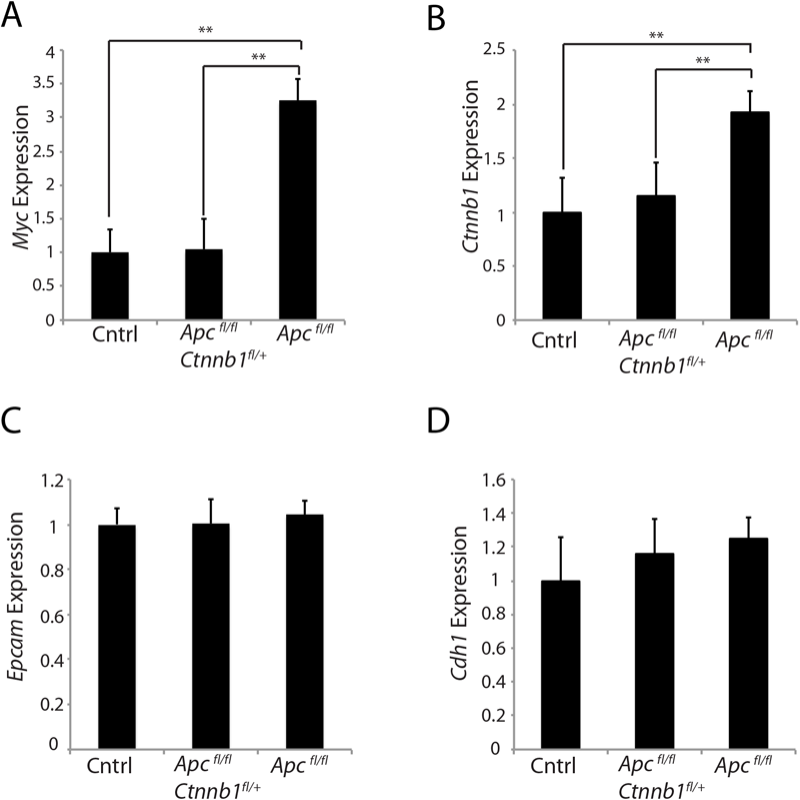
Effects of hemizygous *Ctnnb1* dosage on *Myc* and *Ctnnb1* transcript levels induced by *Apc* inactivation. Gene expression for *Myc* (A), *Ctnnb1* (B), and epithelial cell makers, *Epcam* (C) and *Cdh1* (D), was assessed by qRT-PCR in mouse proximal colon tissues obtained from control *Apc* ^*fl/fl*^ mice (Cntrl), *CDX2P-CreER* ^*T2*^ *Apc* ^*fl/fl*^ *Ctnnb1* ^*fl/+*^ mice (*Apc* ^*fl/fl*^ *Ctnnb1* ^*fl/+*^) and *CDX2P-CreER* ^*T2*^ *Apc* ^*fl/fl*^ mice (*Apc* ^*fl/fl*^) at 20 days after TAM dosing to activate Cre to target alleles. Gene expression was normalized to *β-actin* expression. Two *asterisks* denote *P* < 0.01 in Student's *t* test, and *error bars* denote S.D. (n = 3 for each group).

*Myc* is a well-known β-catenin/TCF-regulated target gene [10, 26], and we found that the strong induction of *Myc* gene expression in mouse colon epithelium seen following *Apc* bi-allelic inactivation was abrogated when *Apc* bi-allelic inactivation occurred concurrently with somatic inactivation of one *Ctnnb1* allele (Fig 5A). Another interesting observation was that *Ctnnb1* transcripts were increased roughly 2-fold in proximal colon tissues following *Apc* bi-allelic inactivation in colon epithelium with wild type *Ctnnb1* gene dosage, compared to the levels of *Ctnnb1* transcript in untargeted colon tissues of *Apc* ^*fl/fl*^ mice (Fig 5B). Hemizygous *Ctnnb1* gene dosage was associated with an inability of *Apc* bi-allelic inactivation to activate *Ctnnb1* transcript levels in proximal colon tissues (Fig 5B). The effects of *Apc* inactivation and *Ctnnb1* gene dosage in mouse colon epithelium on *Myc* and *Ctnnb1* transcript levels did not appear to simply reflect a change in the epithelial cell numbers in *Apc*-mutant colon epithelium, because transcripts for the epithelial markers epithelial cell adhesion molecule (*Epcam*) and E-cadherin (*Cdh1*) were similar in the mouse colon tissues independent of genotype (Fig 5C and 5D). The findings indicate that not only does *Apc* inactivation lead to increased β-catenin protein levels in murine colon tissues, but *Ctnnb1* transcript levels in the colon tissues are also increased by *Apc* inactivation, consistent with an apparent feed-forward mechanism for up-regulation of *Ctnnb1* transcripts following *Apc* inactivation. Because the *Apc* mutation-dependent induction of *Ctnnb1* transcripts in mouse colon epithelium was not seen in the setting of reduced *Ctnnb1* gene dosage, the findings imply that the feed-forward mechanism for *Ctnnb1* induction may require sufficient levels of β-catenin and β-catenin/TCF-dependent transcription.

**Fig 6:**
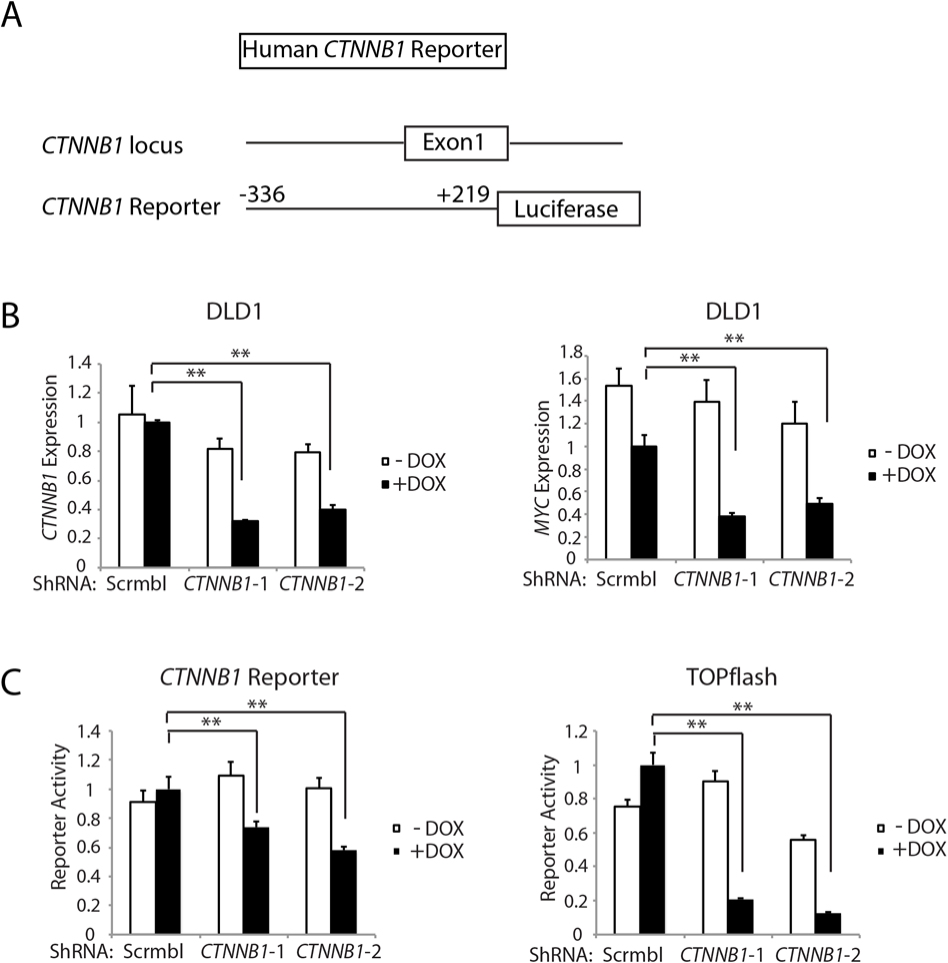
Inhibition of *CTNNB1* transcript levels affects *MYC* transcript levels and *CTNNB1* reporter gene activity in human DLD1 colon cancer cells. (A) Schematic representation of the 5’ region of the human *CTNNB1* gene (top) and the *CTNNB1* reporter gene construct generated (bottom). The *CTNNB1* reporter construct contains *CTNNB1* sequences from −336 to +219 relative to the transcription start site at +1. (B) DLD1 stably transduced with two different doxycycline-inducible shRNAs targeting *CTNNB1* (*CTNNB1*-1 and *CTNNB1*-2) or a non-silencing scramble shRNA (Scrmbl), were treated for 4 days with DOX (“+ DOX”) or a solvent control (“- DOX”). Effects of *CTNNB1* shRNA-mediated inhibition on *CTNNB1* (left) and *MYC* (right) expression are shown. Gene expression was assessed by qRT-PCR and normalized to *HPRT* expression. *Error bars* denote S.D. **P < 0.01 and *P < 0.05. (C) DLD1 cells, stably expressing the two different doxycycline-inducible shRNAs targeting *CTNNB1* or the non-silencing scrambled shRNA (Scrmbl), were transfected with the *CTNNB1* reporter construct, and luciferase activity was measured (bottom left). The cells were treated for 4 days with DOX (“+ DOX”) at 2 μg/ml or a solvent control (“- DOX”). The TOPflash reporter construct was also used to assess effects of *CTNNB1* inhibition on β-catenin/TCF-dependent transcription. The assays were performed in triplicate, mean ± S.D. values are shown, and the control CMV-driven Renilla luciferase-expressing vector was used to correct for differences in transfection efficiency. Two *asterisks* denote *P* < 0.01 in Student's *t* test.

Of interest with regard to a role for β-catenin and β-catenin/TCF transcription in regulating *Ctnnb1* transcription is that chromatin immunoprecipitation (ChIP) studies from the ENCODE project indicate that the TCF4 protein, encoded by the *TCF7L2* gene, is bound in the proximal promoter and exon 1 region of the *CTNNB1* gene in selected cell lines. The mouse *Ctnnb1* and human *CTNNB1* promoter regions lack known TCF family protein consensus binding elements. Nonetheless, to further explore the role of *CTNNB1* transcript and β-catenin levels in regulating *CTNNB1* transcription, we generated a reporter gene construct in which a 555 bp fragment of human *CTNNB1* upstream and exon 1 sequences (-336 to +219 relative to the transcription start site) were cloned upstream of a firefly luciferase sequence (Fig 6A). This region of the *CTNNB1* gene corresponds to the region that the ENCODE ChIP data indicates is occupied by the TCF family member TCF4 in some cell lines. We used two different doxycycline-induced shRNAs against *CTNNB1* to study effects of antagonizing *CTNNB1* endogenous transcript levels in the DLD1 human colon cancer cell line, which harbor APC defects leading to constitutive activation of β-catenin/TCF signaling (Fig 6B). The doxycycline-mediated shRNA-mediated inhibition of *CTNNB1* transcript levels in DLD1 cells also led to marked inhibition of *MYC* expression (Fig 6B). In addition, we found *CTNNB1* reporter luciferase activity was inhibited by about 30–40% by the reduction in *CTNNB1* transcripts in the cells, whereas, the prototypical Wnt/β-catenin/TCF reporter gene construct TOPflash was more robustly inhibited by the reduction in *CTNNB1* levels (Fig 6C). These studies and data complement the primary mouse colon tissue findings presented above by showing that reduction of *CTNNB1* levels in colon cancer cells has a demonstrable effect on *CTNNB1* transcriptional activity.

### The active pool of β-catenin protein and β-catenin-regulated target gene transcription are highly sensitive to changes in β-catenin transcript levels in colon epithelial cells

**Fig 7:**
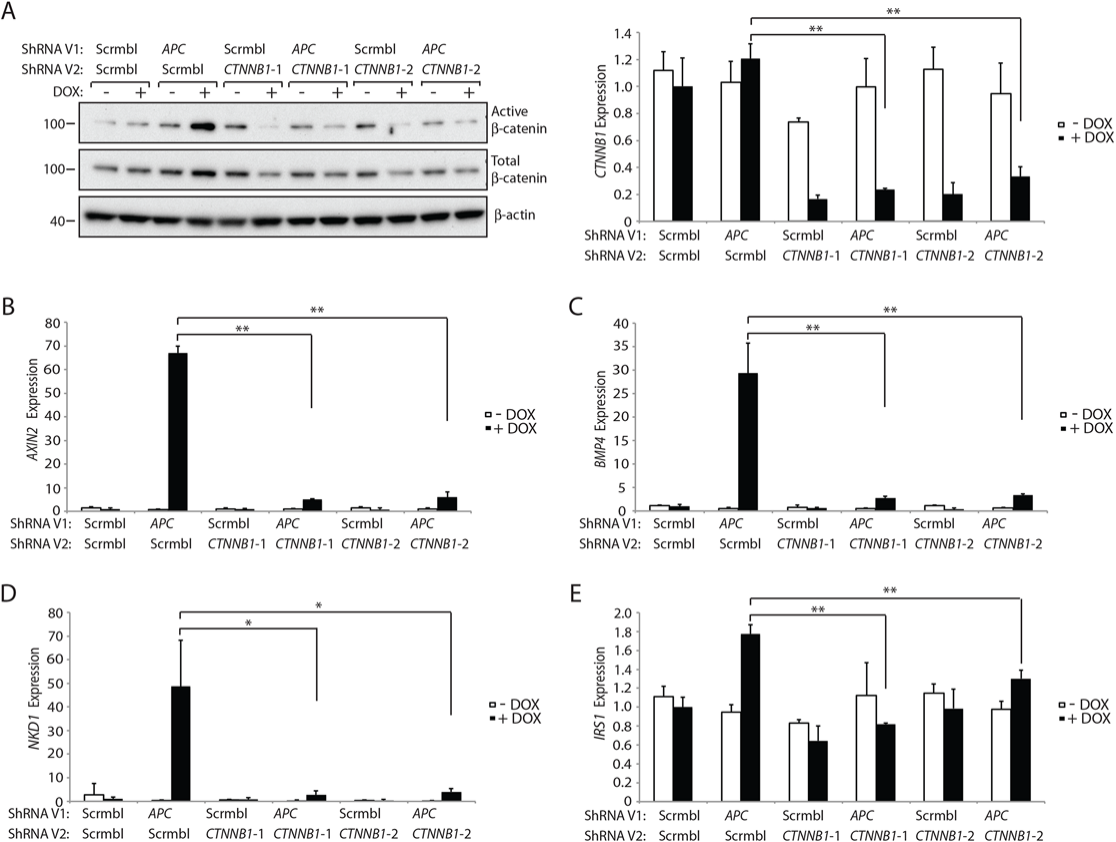
Inhibition of β-catenin expression by CTNNB1 shRNAs dramatically reduces the increase in the active pool of β-catenin and β-catenin/TCF-regulated target gene expression seen following *APC* inhibition in HCECs. HCECs stably transduced with a lentiviral vector (ShRNA V1) expressing an *APC* shRNA or a non-silencing scramble shRNA (Scrmbl) were further transduced with lentiviral vectors (ShRNA V2) driving expression of two different shRNAs targeting *CTNNB1* (*CTNNB1*-1 and *CTNNB1*-2) or a non-silencing scramble shRNA (Scrmbl). Protein and RNA were collected from the HCECs following a 3-day induction of shRNA expression by addition of doxycycline (DOX, “+”) at 2 μg/ml or no DOX-mediated induction (i.e., solvent control “-“). (A) ShRNA-mediated changes in the expression of the total and active (un- or hypophosphorylated) pools of β-catenin in the HCECs were assessed by Western blot analysis for active β-catenin and total β-catenin, with β-actin as a loading and transfer control (left) and by qRT-PCR for *CTNNB1* transcripts normalized to *U6* expression (right). The two *asterisks* denote *P* < 0.01 in Student's *t* test, and *error bars* denote S.D. Analysis of the expression of selected Wnt/β-catenin/TCF-regulated target genes was undertaken in the cell lines with or without DOX-mediated induction of the indicated shRNAs—*AXIN2* (B), *BMP4* (C), *NKD1* (D) and *IRS1* (E), as assessed by qRT-PCR and normalized to *U6* expression. In panels (*B*), (C) and (E), **P < 0.01. In panel (D), *P < 0.05.

Canonical (β-catenin-dependent) Wnt signaling is dependent on increases in the levels and localization of a hypo- or un-phosphorylated pool of β-catenin, which is often termed the “free” or “active” pool of β-catenin in the cytoplasm and nucleus of cells with Wnt pathway activation [27, 28]. Active β-catenin can function as a co-activator for TCF-dependent transcription of endogenous Wnt/β-catenin/TCF target genes [27]. To further address how reduced *CTNNB1* gene expression affects β-catenin protein levels and β-catenin/TCF-regulated target gene induction in colon epithelial cells, we used an shRNA approach to antagonize *CTNNB1* transcript and β-catenin protein levels in colon cell lines. In the immortalized, non-neoplastic human colon epithelial cell (HCEC) line, through use of a doxycycline (DOX)-regulated shRNA against *APC*, we reduced endogenous *APC* gene and protein expression in the cells to less than 10% of control levels (S5 Fig). Following *APC* shRNA induction, the levels of active β-catenin, as detected with a previously described antibody against the hypo-phosphorylated or active form of β-catenin, were significantly increased, whereas only a minor increase in total β-catenin levels was seen (Fig 7A and S6 Fig). Concurrent DOX-mediated induction of the *APC* shRNA and either of the two independent *CTNNB1* shRNAs, which reduced *CTNNB1* transcript levels to about 20–30% of control levels in HCECs, led to dramatic inhibition of the *APC* inactivation-stimulated effects on active β-catenin protein levels, but only modest to moderate reduction in the levels of total β-catenin protein (Fig 7A and S6 Fig). The marked effects of the *APC* and *CTNNB1* shRNA approaches on the active β-catenin pool, with only more modest to moderate effects on total β-catenin levels in HCEC cells were reproducible (S6 Fig). Following *APC* shRNA induction by DOX treatment, expression of multiple β-catenin/TCF-regulated target genes, such as *AXIN2*, *BMP4*, *NKD1* and *IRS1*, was significantly induced in HCECs (Fig 7B–7E). These *APC* shRNA-mediated increases in β-catenin/TCF-regulated target gene expression were almost completely abolished by shRNA-mediated inhibition of β-catenin (Fig 7B–7E). We also studied sub-cellular localization of β-catenin in the HCEC cells following *APC* shRNA induction and combined *APC* and *CTNNB1* shRNA induction by DOX. Consistent with the marked increase in active β-catenin levels following *APC* shRNA induction, we found β-catenin protein mainly accumulated in the cytosol and nucleus of HCECs (S7 Fig). Concurrent induction of both the *APC* and *CTNNB1* shRNAs in HCECs dramatically reduced the levels of β-catenin protein in the nucleus and cytoplasm (S7 Fig), consistent with the notion that the active, signaling pool of β-catenin in the cytoplasm and nucleus is highly sensitive to changes in *Ctnnb1* transcript levels.

The strong inhibitory effect on the active pool of β-catenin compared to that for total β-catenin when *CTNNB1* transcript levels were reduced in HCECs was further studied in three human colon cancer cell lines stably transduced with the two DOX-inducible *CTNNB1* shRNAs. These included a colon cancer cell line with a gain-of-function mutation in *CTNNB1* (HCT116) and two colon cancer cell lines with *APC* loss-of-function mutations (DLD1 and SW480). At 7 days after DOX-induction of the *CTNNB1* shRNAs, in the three colon cancer cell lines, we found moderate (HCT116) to dramatic (DLD1 and SW480) decreases in the active pool of β-catenin protein with only modest changes in total β-catenin protein levels (S8 Fig). Expression of the *CTNNB1* shRNAs led to potent inhibition of the expression of Wnt/β-catenin/TCF-regulated target genes in the DLD1 and HCT116 cells, including *AXIN2*, *BMP4*, *NKD1*, *LGR5*, and *CD44* (S9 Fig).

### *Ctnnb1* hemizygous inactivation does not affect *Apc*- and *Pten*-mutation-dependent mouse ovarian endometrioid adenocarcinoma (OEA) development

**Fig 8:**
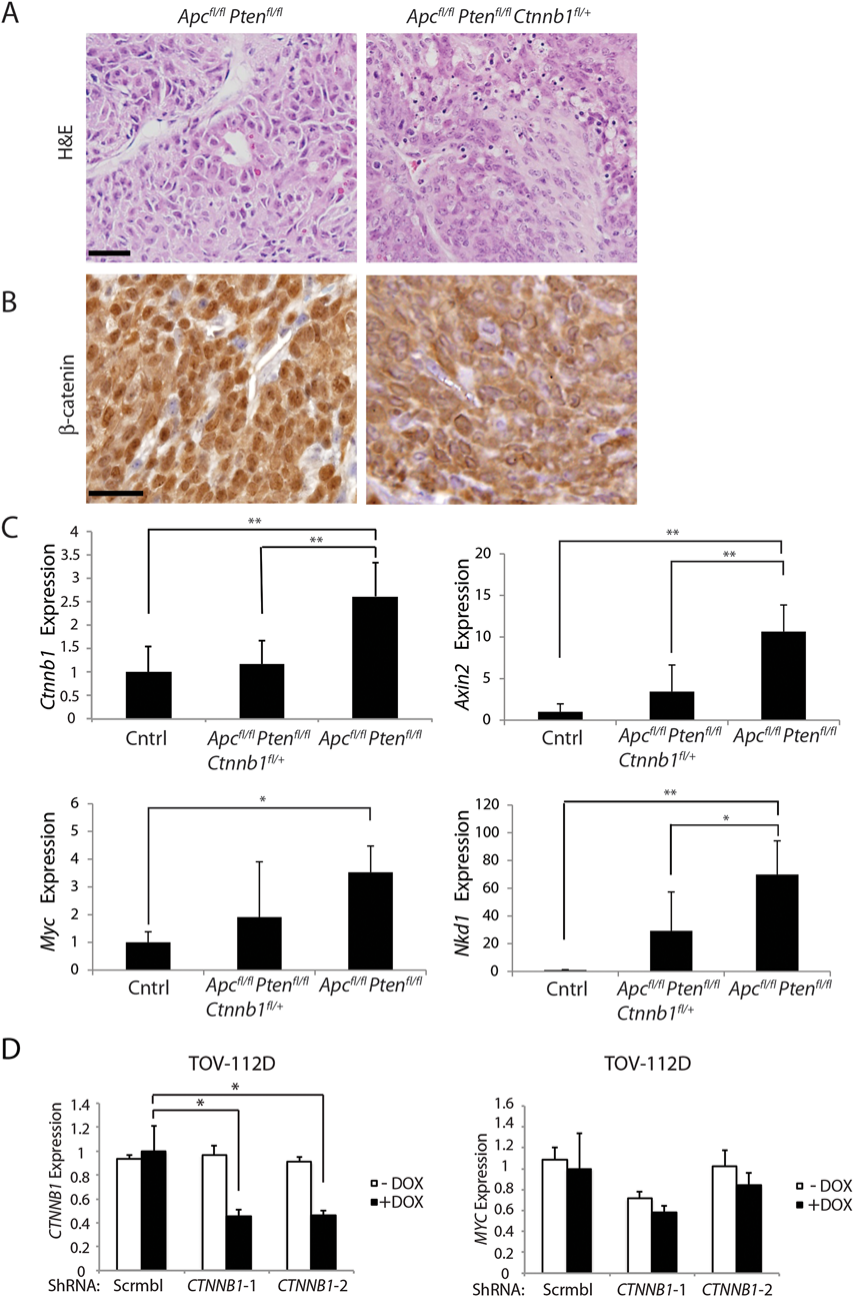
Heterozygous *Ctnnb1* inactivation does not prevent mouse ovarian endometrioid adenocarcinoma (OEA) development and the reduction in *Ctnnb1 dosage or CTNNB1* transcripts shows differential effects on selected β-catenin/TCF target genes. (A) Representative photomicrographs of H&E stained ovarian cancer tissue sections from *Apc* ^*fl/fl*^ *Pten* ^*fl/fl*^, and *Apc* ^*fl/fl*^ *Pten* ^*fl/fl*^ *Ctnnb1* ^*fl/+*^ mice show similar morphologic characteristics independent of *Ctnnb1* gene dosage, with areas of glandular, overtly epithelial differentiation admixed with more poorly differentiated spindle-cell areas. Scale bars, 100 μm. (B) Representative photomicrographs of β-catenin immunohistochemical staining in tumors from *Apc* ^*fl/fl*^ *Pten* ^*fl/fl*^ mice, showing strong nuclear β-catenin translocation and moderate cytoplasmic staining, whereas tumors from *Apc* ^*fl/fl*^ *Pten* ^*fl/fl*^ *Ctnnb1* ^*fl/+*^ mice showed weak nuclear and moderate cytoplasmic β-catenin staining. Scale bars, 25 μm. (C) cDNAs were obtained from murine normal ovaries (Cntrl, n = 4) and OEAs from *Apc* ^*fl/fl*^ *Pten* ^*fl/fl*^ *Ctnnb1* ^*fl/+*^ (n = 6), and *Apc* ^*fl/fl*^ *Pten* ^*fl/fl*^ (n = 6) mice following AdCre injection. Transcript levels of *Ctnnb1*, *Axin2*, *Myc* and *Nkd1* were measured by qRT-PCR analysis and normalized with β-actin. The gene expression levels were set to 1 for control (Cntrl) group. *Error bars* denote S.D. **P < 0.01 and *P < 0.05. (D) TOV-112D cells (the human OEA-derived cell line harboring β-catenin gain of function mutation), stably transduced with two different doxycycline-inducible shRNAs targeting *CTNNB1* (*CTNNB1*-1 and *CTNNB1*-2) or a non-silencing scramble shRNA (Scrmbl), were treated for 4 days with DOX (“+ DOX”) or a solvent control (“- DOX”). *CTNNB1* shRNA-mediated effects on *CTNNB1* (left) and *MYC* (right) gene expression are shown. Gene expression was assessed by qRT-PCR and normalized to *HPRT* expression. *Error bars* denote S.D. *P < 0.05 in Student's *t* test.

To assess the role of β-catenin function in another *Apc* mutation-dependent tumor model, we explored the role of *Ctnnb1* gene dosage in a mouse model of ovarian endometrial adenocarcinoma (OEA) arising from bi-allelic inactivation of both the *Apc* and *Pten* genes [29]. Prior studies have shown that the Wnt/β-catenin/Tcf signaling pathway is deregulated by mutations in 16%–38% of human OEAs, and *PTEN* mutations are often seen in the OEAs with Wnt pathway mutations [29–32]. In the mouse OEA model, tumors are initiated by conditional inactivation of the *Apc* and *Pten* genes following injection of AdCre into the right ovarian bursa of *Apc* ^*fl/fl*^ *Pten* ^*fl/fl*^ mice [29]. Interestingly, in both *Apc* ^*fl/fl*^ *Pten* ^*fl/fl*^ mice and *Apc* ^*fl/fl*^ *Pten* ^*fl/fl*^ *Ctnnb1* ^*fl/+*^ mice, adenocarcinomas morphologically similar to human OEAs formed following AdCre injection, with 100% penetrance and no difference in tumor latency between mice with two wild type *Ctnnb1* alleles or one wild type and one floxed *Ctnnb1* allele (Table 1). In addition, no significant differences in survival rates, tumor volumes, and rates of liver metastasis were found between AdCre-injected *Apc* ^*fl/fl*^ *Pten* ^*fl/fl*^ mice and *Apc* ^*fl/fl*^ *Pten* ^*fl/fl*^ *Ctnnb1* ^*fl/+*^ littermates (Table 1), and OEAs arising in both lines of mice shared similar histological features and immunohistochemical staining patterns for cytokeratin-8 (CK8), E-cadherin and α-inhibin (Fig 8A and Table 1). Efficient Cre-mediated deletion of *Ctnnb1* and *Apc* was confirmed in tumors from these mice, and no OEAs arose in the AdCre-injected right ovaries in *Apc* ^*fl/fl*^ *Pten* ^*fl/fl*^ *Ctnnb1* ^*fl/fl*^ mice, indicating OEAs could not arise from cells completely lacking β-catenin.

**Table 1:** Phenotypic features in an *Apc-* and *Pten-* mutant mouse ovarian adenocarcinoma model with respect to *Ctnnb1* gene dosage.

	*Apc^fl/fl^Pten^fl/fl^*	*Apc^fl/fl^Pten^fl/fl^Ctnnb1^fl/+^*	P value*
**n**	6	10	
**Median survial**	79	77	0.5627
**Tumor Penetrance**	6/6	10/10	1.0000
**Tumor Volume**	4.318 ± 0.8177	6.510 ± 0.7193	0.0721
**Liver Metastasis**	2/6	4/10	1.0000
**Histology**	Poorly differentiated adenocarcinoma	Poorly differentiated adenocarcinoma	
**Immunostaining**			
β-catenin	Strong nuclear, moderate cytoplasmic	Weak nuclear and moderate cytoplasmic	
CK8	+	+	
E-cad	+	+	
α-inhibin	-	-	

* Kaplan-Meier survival curves were compared by log-rank (Mantel-Cox) test. P <0.05 was statistically significant

The findings on the lack of a demonstrable effect of *Ctnnb1* hemizygous gene dosage in the mouse OEA model contrast with the findings above, where *Apc*-mutation-dependent polyposis in colon epithelium was dramatically suppressed by *Ctnnb1* hemizygous inactivation. Nonetheless, similar to the situation in mouse colon, based on immunohistochemical staining, the presumptive Wnt pathway signaling-competent pool of β-catenin in the nucleus and cytoplasm was significantly reduced in the OEAs in *Apc* ^*fl/fl*^ *Pten* ^*fl/fl*^ *Ctnnb1* ^*fl/+*^ mice compared to OEAs in the *Apc* ^*fl/fl*^ *Pten* ^*fl/fl*^ mice (Fig 8B). We also examined the β-catenin/TCF-mediated gene transcription in the OEAs arising in the *Apc* ^*fl/fl*^ *Pten* ^*fl/fl*^ mice and *Apc* ^*fl/fl*^ *Pten* ^*fl/fl*^ *Ctnnb1* ^*fl/+*^ mice. Consistent with the β-catenin dosage-dependent effects of *Ctnnb1* transcripts seen in mouse *Apc*-mutant colon tissues described above, *Ctnnb1* transcripts were significantly reduced in the OEAs arising in *Apc* ^*fl/fl*^ *Pten* ^*fl/fl*^ *Ctnnb1* ^*fl/+*^ mice compared to the OEAs in *Apc* ^*fl/fl*^ *Pten* ^*fl/fl*^ mice (Fig 8C). Interestingly, although the *Ctnnb1* hemizygous state in the *Apc*- and *Pten*-mutant OEAs markedly suppressed the induction of some β-catenin/TCF-regulated target genes, such as *Axin2 and Nkd1* (Fig 8C), hemizygous *Ctnnb1* function did not abrogate induction of *Myc* transcripts in the OEAs (Fig 8C). Therefore, our findings showing that *Ctnnb1* hemizgyous state did not prevent development of *Apc*- and *Pten*-mutant OEAs even though there was a reduction in β-catenin levels and expression of some β-catenin/TCF-regulated genes suggest that retention of *Myc* induction in OEAs with hemizygous *Ctnnb1* function, but not in *Apc*-deficient colon epithelium with hemizygous *Ctnnb1* function, may be a contributing factor in the observed differences in tumor development in the two tissues. We also studied the consequences of shRNA-mediated inhibition of *CTNNB1* on *MYC* gene expression in the TOV112D human ovarian endometrioid carcinoma cell line that harbors a *CTNNB1* oncogenic mutation leading to β-catenin/TCF dysregulation [33]. We found that doxycycline-mediated induction of the two *CTNNB1* shRNAs in TOV112D cells reduced *CTNNB1* levels to about 50% of baseline (Fig 8D), but no statistically significant effect on *MYC* transcript levels was seen.

## Discussion

Mutations inactivating the *APC* tumor suppressor gene are believed to be critical initiating lesions in the majority of colon adenomas and carcinomas [6, 34, 35]. *APC* mutations are likely key contributing factors in the development of some other cancer types, including a subset of human OEAs. The best understood function of the APC protein is to act as a component of a phosphorylation- and ubiquitination-dependent destruction complex that regulates the free or active pool of β-catenin. This pool of β-catenin functions as a regulator of TCF transcription in the Wnt pathway signaling [1]. In the studies described above, we assessed the effects of *Ctnnb1* gene dosage on *Apc* mutation-instigated tumorigenesis in mouse genetically engineered colon and ovarian tumor models and in cultured cells. We found the florid polyposis phenotype resulting from somatic *Apc* bi-allelic inactivation in mouse colon epithelium is potently inhibited by concurrent somatic inactivation of one *Ctnnb1* allele. The few polyps arising in *CDX2P-CreER* ^*T2*^ *Apc* ^*fl/fl*^ *Ctnnb1* ^*fl/+*^ mice were found to have escaped *Ctnnb1* targeting, though Cre-mediated somatic inactivation of both *Apc* alleles occurred in the lesions, likely reflecting strong positive selection for maintenance of wild type *Ctnnb1* gene dosage for *Apc*-mutant colon adenomas to arise and persist. In contrast to the situation in colon epithelium, in a mouse model of the human OEAs that harbor inactivating mutations in the *APC* and *PTEN* genes, we found that, regardless of whether the mice had two wild type *Ctnnb1* alleles or one wild type and one targeted *Ctnnb1* allele, adenocarcinomas morphologically similar to human OEAs formed with 100% penetrance and no differences in latency, size, morphology, or metastatic potential of the lesions arising from AdCre-mediated targeting of the *Apc* and *Pten* genes.

In-depth mutational analyses of the germline and somatic mutations in adenomas arising in patients with FAP led to the proposal there was strong biological selection for a “just-right” level of β-catenin signaling that would be optimal for tumor formation [36]. Some prior studies have used genetic approaches to study experimentally the effects of *Ctnnb1* gene dosage on *Apc* mutation-dependent tumorigenesis in the small intestine, liver, and mammary gland in mouse models [14, 15]. The earlier work indicated that the *Ctnnb1* ^*+/-*^ constitutional hemizygous state can inhibit intestinal and liver tumorigenesis in mice carrying *Apc* mutations. In mammary gland tumorigenesis, tumorigenesis was enhanced in *Apc* ^*1638N*^ *Ctnnb1* ^*+/-*^ mice relative to *Apc* ^*1638N*^ *Ctnnb1* ^*+/+*^ mice, perhaps because *Ctnnb1* functions as a tumor suppressor gene in *Apc* ^*1638N*^ mammary gland tumors via β-catenin’s role in E-cadherin-dependent tumor suppression [14]. While our findings in a mouse *Apc* mutation-dependent colon tumorigenesis model are consistent with the prior work on *Apc* mutation-instigated small intestine and liver tumorigenesis, some significant differences in the studies should be noted. The prior work emphasized models where the mice carried constitutional mutations in one *Apc* allele and tumors arose following stochastic loss or inactivation of the remaining wild type *Apc* allele. In addition, mice in the prior small intestine work were constitutionally hemizygous for *Ctnnb1*. In our *Apc* mutation-dependent colon tumorigenesis model, both *Apc* alleles are somatically inactivated in colon epithelium by Cre-mediated targeting, and the *Ctnnb1* hemizygous deficiency state was also somatically generated only in the colon epithelial cells by Cre-mediated targeting. In addition, our OEA model work contrasts with the prior published work, as it also relies on somatic targeting of *Apc* and *Ctnnb1*. The OEA results also differ from our own colon tumorigenesis results, as the findings indicate *Ctnnb1* hemizygous gene dosage had no demonstrable effect on cancer latency, size, or morphology or the metastatic potential of mouse OEAs arising from combined somatic inactivation of *Apc* and *Pten*.

Besides highlighting tissue-specific differences for *Ctnnb1* gene dosage in *Apc* mutation-instigated colon and ovarian tumorigenesis, our studies and data have provided several unique and in-depth insights into cell and tissue mechanisms by which *Ctnnb1* gene dosage likely contributes to *Apc* mutation-dependent phenotypes in mouse colon epithelium. Inactivation of one *Ctnnb1* allele markedly inhibited the increases in β-catenin cytoplasmic and nuclear levels that result from bi-allelic *Apc* inactivation in mouse colon epithelium. In turn, there was strikingly attenuated expression of key β-catenin/TCF-regulated target genes, including those encoding the EphB2/B3 receptors, and the stem cell markers Lgr5, Msi1, and Hopx. Of significant interest in terms of a likely key mechanisms through which *Ctnnb1* gene dosage inhibits adenoma formation, the inability of the *Apc*-mutant colon epithelial cells to up-regulate and alter the crypt (high)-surface (low) gradient of EphB2/B3 expression appears to restrict high levels of EphB2/3 expression to the crypt base. Activated β-catenin/TCF transcription has been implicated in repression of ephrinB expression [11, 23]. We found that the robust ephrinB1/B2 expression seen in the upper two-thirds of normal colon crypts as well as in the normal colon surface epithelium was maintained in *Apc*-mutant crypts when one *Ctnnb1* allele was inactivated. In contrast, *Apc*-mutant crypts with intact *Ctnnb1* dosage markedly down-regulated ephrinB1/B2 expression and dramatically upregulated and expanded EphB2/B3 expression throughout the colon crypts. Because the EphB and ephrin molecules mediate critical repulsive interactions in intestinal crypts, the maintenance of the normal inverse EphB/ephrinB gradient from crypt base to cell surface in *Apc*-mutant crypts where one *Ctnnb1* allele is inactive restricts the expansion of the Lgr5-positive crypt stem cell pool and the crypt fission/branching that would result from unrestrained crypt stem cell expansion and altered migration [11, 23]. As a result of the preservation of the inverse gradient of EphB/ephrinB expression in *Apc* mutant crypts with reduced *Ctnnb1* dosage, stem cell expansion and the dysplastic and adenomatous changes induced by *Apc* inactivation in colon epithelium are potently inhibited, even though Lgr5 and some other stem cell marker genes are modestly increased in expression in the targeted crypts of *CDX2P-CreER* ^*T2*^ *Apc* ^*fl/fl*^ *Ctnnb1* ^*fl/+*^ mice relative to crypts in normal mice.

Our findings showing that *Ctnnb1* transcripts are up-regulated in *Apc*-mutant mouse colon epithelium as well as in *Apc*-mutant mouse OEAs, together with our findings that *Ctnnb1* hemizygous gene dosage inhibited *Apc* mutation-dependent *Ctnnb1* transcript induction in colon and ovarian tumor models imply that the *Ctnnb1* gene is subject to feed-forward activation by β-catenin levels and β-catenin/TCF-regulated transcription. Of interest with regard to a role for β-catenin and β-catenin/TCF transcription in regulating *Ctnnb1* transcription are chromatin immunoprecipitation (ChIP) studies from the ENCODE project reporting that the TCF4 protein, encoded by the *TCF7L2* gene, is bound in the promoter region of the *CTNNB1* gene in selected cell lines. Based on our mouse colon tissue studies and the ENCODE project findings, we generated a *CTNNB1* reporter gene construct containing 555 bp of human *CTNNB1* upstream and exon 1 sequences and found that shRNA-mediated inhibition of *CTNNB1* endogenous gene expression in the *APC*-mutant DLD1 human colon cancer cell line led to inhibition of the activity of the *CTNNB1* reporter gene. These data demonstrate that *CTNNB1* transcript levels affected *CTNNB1* transcription in colon cancer cells. The lack of known TCF consensus element binding sites in the mouse *Ctnnb1* and human CTNNB1 promoter regions currently limits support for the argument that β-catenin/TCF transcription directly regulates *Ctnnb1* transcription, though further studies to address the point will need to be pursued.

In contrast to the near complete abrogation of *Myc* induction in *Apc*-mutant colon epithelium with one *Ctnnb1* allele, *Myc* induction was retained in the *Apc*-mutant mouse OEAs with one functional *Ctnnb1* allele. Of note, in prior studies, it has been shown that hemizyous inactivation of *Myc* dramatically inhibited *Apc* mutation-induced small intestine tumor phenotypes, but not *Apc* mutation-induced effects on liver cell proliferation and size [37, 38]. Hence, the findings from our work and these prior studies [37–39] highlight *Myc* as perhaps one of the key β-catenin/TCF-regulated genes with tissue-specific differences in its regulation by β-catenin/TCF that may account for why *Ctnnb1* hemizygous state abrogates *Apc* mutation-induced effects in some tissues (e.g., small intestine and colon epithelium) but not in other tissues (e.g., liver and ovarian epithelium). The identification of a possible feed-forward mechanism for β-catenin and β-catenin/TCF transcription in regulating *Ctnnb1* transcript levels following *Apc* inactivation are also potentially interesting with regard to *Myc*, because the ENCODE project work also indicates that the Myc protein is bound in the promoter and intron one regions of the *Ctnnb1* gene in selected cell lines. As such, β-catenin/TCF transcription may cooperate in some fashion with Myc, itself encoded by a β-catenin/TCF target gene, in a more complex feed-forward loop to activate *Ctnnb1* transcription in certain cell types when *Apc* is inactivated.

Further studies are needed to better understand the details of the apparent feed-forward mechanisms through which β-catenin and β-catenin/TCF transcription may regulate *Ctnnb1/CTNNB1* transcription in the setting of *Apc/APC* inactivation. Besides *Myc*, other β-catenin/TCF target genes may also be differentially regulated in a tissue- and context-dependent fashion, perhaps contributing in some fashion to the tissue-specific differences of *Ctnnb1* hemizygous gene dosage on *Apc* mutation-instigated tumorigenesis observed. In addition, the basis for the dramatic changes in the free or active pool of β-catenin protein relative to the more modest effects on total β-catenin protein levels when Ctnnb1/*CTNNB1* transcript levels are reduced in colon epithelial cells with Wnt pathway dysregulation remains to be elucidated. Nonetheless, our findings highlight the possibility that novel approaches and/or agents that can reduce *CTNNB1* transcript levels and/or the free pool of β-catenin protein might have quite dramatic effects on the development and perhaps persistence of neoplastic cells with Wnt pathway defects.

## Materials and Methods

### Mice

To target *Apc* and/or *Ctnnb1* alleles in colon tissues, *CDX2P-G22Cre* transgenic mice [16], or *CDX2P-CreER* ^*T2*^ transgenic mice [17], or *Lgr5-EGFP-IRES-CreER* ^*T2*^ (*B*6.129P2-*Lgr5* ^tm1(cre/ERT2)Cle^/J) transgenic mice [8] (The Jackson Laboratory, Bar Harbor, ME), were first intercrossed with mice homozygous for *Apc* targeted alleles (*Apc* ^*fl/fl*^, 580S) [40] and *Ctnnb1-*targeted alleles (*Ctnnb* ^*fl/fl*^, B6.129-*Ctnnb1* ^*tm2Kem*^/KnwJ) [41]. The resulting Cre positive *Apc* ^*fl/+*^ *Ctnnb1* ^*fl/+*^ mice were then crossed to *Apc* ^*fl/fl*^ mice in order to target two alleles of *Apc* and one allele of *Ctnnb1* (*Apc* ^*fl/fl*^ *Ctnnb1* ^*fl/+*^) or only alleles of *Apc* (*Apc* ^*fl/fl*^), respectively. The Cre positive *Apc* ^*fl/fl*^ *Ctnnb1* ^*fl/+*^ and *Apc* ^*fl/fl*^ littermates were compared and the Cre negative littermates served as normal control. The *CDX2P-CreER* ^*T2*^ *Lgr5-EGFP-IRES-CreER* ^*T2*^ *Apc* ^*fl/fl*^ compound mice *or CDX2P-CreER* ^*T2*^ *Lgr5-EGFP-IRES-CreER* ^*T2*^ *Apc* ^*fl/fl*^ *Ctnnb1* ^*fl/+*^ compound mice were constructed by crossing *Lgr5-EGFP-IRES-CreER* ^*T2*^ *Apc* ^*fl/fl*^ mice to *CDX2P-CreER* ^*T2*^ *Apc* ^*fl/fl*^ mice and *CDX2P-CreER* ^*T2*^ *Apc* ^*fl/fl*^ *Ctnnb* ^*fl/fl*^ littermates, respectively. To assess *Cre*-mediated recombination or Wnt signaling in colon epithelium, mice carrying the *Gt*(*ROSA*)*26Sor ^tm1(EYFP)Cos^/J* reporter allele (EYFP) [42] or the *B6*.*129P2-Axin2* ^*tm1Wbm*^ */J* allele (Axin2-LacZ) [43] (The Jackson Laboratory) were bred into *CDX2P-CreER* ^*T2*^ *Apc* ^*fl/fl*^ mice *or CDX2P-CreER* ^*T2*^ *Apc* ^*fl/fl*^ *Ctnnb1* ^*fl/+*^ mice. To assess the role of β-catenin function in another *Apc* mutation-dependent mouse tumor model, we used the previously described mouse model of ovarian endometrioid adenocarcinoma (OEA), arising from bi-allelic inactivation of both the *Apc* and *Pten* genes (*Apc* ^*fl/fl*^ *Pten* ^*fl/fl*^) [29]. To introduce the floxed *Ctnnb1* allele, *Ctnnb* ^*fl/fl*^ mice were first crossed to *Apc* ^*fl/fl*^ *Pten* ^*fl/fl*^ mice to generate *Apc* ^*fl/+*^ *Pten* ^*fl/+*^ *Ctnnb* ^*fl/+*^ mice, and then *Apc* ^*fl/+*^ *Pten* ^*fl/+*^ *Ctnnb* ^*fl/+*^ mice were bred to *Apc* ^*fl/fl*^ *Pten* ^*fl/fl*^ mice to generate *Apc* ^*fl/fl*^ *Pten* ^*fl/fl*^, and *Apc* ^*fl/fl*^ *Pten* ^*fl/fl*^ *Ctnnb* ^*fl/+*^ mice. All mice were on a mixed C57BL/6 and 129 background, which were backcrossed to C57BL/6 mice for at least 10 generations, except the EYFP reporter mice and the *CDX2P-CreER* ^*T2*^ transgenic mice, which were backcrossed for 7 and 3 generations, respectively. All experimental compound mice were on a mixed C57BL/6 and 129 background, and littermates with similar genetic background and different genotypes were used for comparison (see breeding scheme above). Animal husbandry and experimental procedures were carried out under approval from the University Committee on Use and Care of Animals, University of Michigan and according to Michigan state and US federal regulations. All the mice were housed in specific-pathogen free (SPF) conditions. After weaning, rodent 5001 chow and automatically supplied water were provided *ad libitum* to mice. Animals were euthanized and analyzed at the specified time points, based on particular study design parameters or defined humane treatment and euthanasia guidelines.

### Cell lines and RNA interference

Human colonic epithelial cells (HCEC) [44] were kindly provided by Dr. Jerry Shay (UT Southwestern Medical School, Dallas, TX) and routinely grown on media made up with Dulbecco's modified Eagle's medium (DMEM; Life Technologies, Grand Island, NY) and medium 199 (Thermo Scientific HyClone, Waltham, MA) at the ratio of 4:1, supplemented with EGF (25 ng/mL) (PeproTech, Inc, Rocky Hill, NJ), insulin (10 μg/mL, Life Technologies), hydrocortisone (1 μg/mL), transferrin (2 μg/mL), sodium selenite (5 nm) (all from Sigma-Aldrich, St Louis, MO), and 2% cosmic calf serum (Thermo Scientific HyClone). Cells were cultured on Primaria dishes (BD Biosciences, San Jose, CA) or chamber slides (Lab-Tek II, Vernon Hills, IL) and grown in 2% oxygen and 7% carbon dioxide. HCEC cells were infected with a TRIPZ inducible lentiviral vector (GE Dhamacon, Lafayette, CO) carrying a shRNA against *APC* (targeting sequence: 5’-CAAATCATATGGATGATAA-3’) or a non-silencing scramble shRNA (Scrmbl). Cells were selected with 1μg/mL of puromycin (Sigma-Aldrich) for 5 days. The resulting stable cell lines (HCEC/*APC* shRNA or HCEC/Scrmble) were further transduced with TRIPZ lentiviruses driving expression of two different shRNAs targeting *CTNNB1* (*CTNNB1*-1 and *CTNNB1*-2; targeting sequence for *CTNNB1*-1: 5’-TGGGTGGTATAGAGGCTCT-3’; and targeting sequence for *CTNNB1*-2: 5’- AGCTGATATTGATGGACAG-3’) or a non-silencing scramble shRNA (Scrmbl). Human colon cancer cell lines, HCT116, SW480, and DLD1, and human OEA-derived cell line, TOV-112D, were grown in 5% CO_2_ with DMEM containing 10% fetal bovine serum and penicillin/streptomycin. HCT116, SW480, DLD1 and TOV-112D cells stably expressing the shRNAs targeting *CTNNB1* (*CTNNB1*-1 and *CTNNB1*-2) or a non-silencing scramble shRNA (Scrmbl) were made in the same way as HCEC cells. Expression of shRNAs was induced by incubation of cells with doxycycline (DOX; Sigma-Aldrich) at 2 μg/ml or a solvent control for 3 days (for HCEC cells) or 7 days (for HCT116, SW480, and DLD1 cells). The degree of inhibition of the shRNAs on *APC* transcripts and protein and *CTNNB1* transcripts and the respective β-catenin protein was assessed by qRT-PCR and Western blotting assays.

### Plasmids

DNA fragment containing human *CTNNB1* sequences from −336 to +219 relative to the transcription start site was obtained by PCR amplification of genomic DNA, and was subcloned upstream from the luciferase reporter gene in the pGL3Basic reporter vector (Promega, Madison, WI), using the MluI and XhoI sites. The forward primer for generating the CTNNB1 reporter construct was 5′-ACGCGTGCTGCTCTCCCGGTTCG -3′; the reverse primer for generating the CTNNB1 reporter construct was 5′- CTCGAGCAGGGGAACAGGCTCCTC-3′.

### Tamoxifen (TAM) treatment and AdCre injection

Mice with the *CDX2P-CreER* ^*T2*^ transgene or *Lgr5-EGFP-IRES-CreER* ^*T2*^ were injected intraperitoneally with TAM (Sigma-Aldrich) dissolved in corn oil (Sigma-Aldrich). For two TAM daily dosing, we used 150mg/kg weight per dose; for three consecutive daily doses, we administered TAM at 100mg/kg weight per dose. Mice were injected with TAM at 2- to 3-months of age. For OEA induction, 5 x 10^7^ plaque-forming units of replication-incompetent recombinant adenovirus expressing Cre recombinase (AdCre, from the University of Michigan’s Vector Core) were injected into the right ovarian bursal cavities of 6–10 week old female mice as previously described [29].

### Immunohistochemistry, immunofluorescence and β-gal analysis

Mouse tissues were prepared for paraffin-embedding or cryosectioning as described previously [16]. For assessment of cell proliferation, mice were pulsed with 5-bromo-2-deoxyuridine (BrdU; Sigma-Aldrich) for 1 hr before euthanasia. Sections of paraffin-embedded human or mouse tissues were subjected to immunohistochemical analysis as previously described [45]. The following primary antibodies were used for immunohistochemical analysis with sections of paraffin-embedded tissues: mouse anti-BrdU (1:500; BD Biosciences); rabbit anti-lysozyme (1:2000; Dako, Carpinteria, CA); mouse anti-β-catenin (1:800; BD Biosciences); rat anti-CK8 (1:100, The Developmental Studies Hybridoma Bank, Iowa City, IA); goat anti-E-cadherin (1:100, R&D Systems, Minneapolis, MN); mouse anti-α-inhibin (1:200, Bio-Rad Laboratories, Inc., Raleigh, NC). For BrdU staining, tissue sections were treated with 2N HCl at 37°C for 30 min after performing antigen retrieval with citrate buffer (pH 6.0, Biogenex, San Ramon, CA). For immunofluorescence using frozen sectioned tissues, mouse colon and intestinal tissues were fixed in 4% paraformaldehyde (PFA) overnight, cryo-protected and frozen in O.C.T. (Fisher HealthCare, Houston, TX 77038). Standard immunofluorescence staining was performed on 6-μm frozen sections with rabbit anti-lysozyme antibody (1:1000; Dako). For immunofluorescence using paraffin-embedded tissues, the following primary antibodies were used: rabbit anti-lysozyme (1:1000; Dako), rabbit anti-Sox9 (1:200; Millipore, Temecula, CA), rat anti-Msi1 (1:500; a gift from Dr. Hideyuki Okano [46, 47]), goat anti-EphB2 (1:100; R&D Systems), goat anti-EphB3 (1:100; R&D Systems), goat anti-ephrinB1 (1:200; R&D Systems), goat anti-ephrinB2 (1:100; R&D Systems), mouse anti-α-tublin (1:1000; Sigma-Aldrich), and rabbit anti-Crb3 (1:1000; kindly provided by Dr. Benjamin Margolis at University of Michigan). The secondary antibodies used were Alexa fluor 488-conjugated donkey anti-goat, Alexa fluor 488-conjugated donkey anti-rabbit, Alexa fluor 488-conjugated goat anti-rabbit, Alexa fluor 594-conjugated goat anti-mouse, Alexa fluor 488-conjugated goat anti-mouse, Alexa fluor 594-conjugated goat anti-rabbit, and Alexa fluor 488-conjugated goat anti-rat (Molecular Probes, Life Technologies, Carlsbad, CA), diluted at 1:1000. DNA was labeled by Hoechst 33342 (Molecular Probes, Life Technologies) by adding to the washing buffer at 5 μg/ml. β-gal analysis for mouse with Axin2-LacZ reporter was performed as described previously [16].

### Terminal deoxynucleotidyl transferase (TdT)-mediated dUTP nick end-labeling (TUNEL) assay

To assess apoptosis, TUNEL assays were undertaken using 4-μm sections of formalin-fixed, paraffin-embedded mouse colon tissues, after the tissue sections were deparaffinized, rehydrated and treated with 20 μg/ml protease K (Roche Applied Sciences, Indianapolis, IN) at 37°C for 15 min. The nicked DNA was labeled by using terminal transferase (TdT) (New England Biolabs, Ipswich, MA) and Biotin-16-UTP (Roche Applied Sciences) according to the manufacturer’s recommendation. The signal was detected by using the Vectastain ABC kit (Vector Laboratories, Burlingame, CA) according to the manufacturer’s suggestion.

### Mitotic spindle axis assessment

The spindle angles were defined by the orientation of mitotic spindles, based on α-tubulin staining, relative to the most adjacent apical membrane, as indicated by Crb3 staining. The mitotic spindle axis angle relative to the planar axis of the cells (defined by the most adjacent apical membrane) was measured by ImageJ (NIH).

### Western blot analysis

Western blot analyses on lysates from HCEC, HCT116, SW480, DLD1 and TOV-112D cells were performed as described [45]. The following antibodies were used: mouse anti-active β-catenin (1:2000; Millipore, Temecula, CA), mouse anti-total β-catenin (1:10,000; BD Biosciences), rabbit anti-APC (clone C-20, 1:1000; Santa Cruz Biotechnology, Santa Cruz, CA), mouse anti-APC (clone Ab-5, 1:1000; Millipore), and mouse anti-β-actin (1:10,000; Sigma). The density of Western blotting bands was quantified using AlphaImager HP system (ProteinSimple, San Jose, CA).

### Quantitative reverse transcription (RT)-PCR (qRT-PCR)

cDNA was synthesized using a high capacity cDNA reverse transcription kit (Applied Biosystems, Foster City, CA). qRT-PCR was performed with an ABI Prism 7300 Sequence Analyzer using a SYBR green fluorescence protocol (Applied Biosystems). See S1 Table for primer sequences used in qRT-PCR.

### Reporter gene assays

DLD1 cells, stably expressing two different doxycycline-inducible shRNAs targeting *CTNNB1* (*CTNNB1*-1 and *CTNNB1*-2) or a non-silencing scramble shRNA (Scrmbl), were treated for 4 days with DOX at 2 μg/ml or a solvent control. At second day during DOX treatment, cells were plated in 35-mm six-well plates. After 12—24h cells were then transfected with 0.5 μg of *CTNNB1* reporter or TOPflash, 1 μg of pCDNA3 (Invitrogen) and 0.05 μg of PRL-CMV *Renilla* luciferase reporter vector (Promega) using Mirus *Trans*IT-LT1 transfection reagent (Mirus Bio, Madison, WI) according to the manufacturer's protocol. Cells were harvested 45h later and luciferase activities were measured using a Dual-luciferase kit and GloMax-Multi Detection System from Promega.

### Statistical analysis

All data for qRT-PCR were evaluated by Student's t test and asterisks denote significance with *P <* 0.05. Error bars denote standard deviations (S.D.). Kaplan-Meier survival curves were compared by log-rank (Mantel-Cox) test. Chi-Square test was used to determine significance when mitotic spindle angles were compared among mice with different genotypes. *P* < 0.05 is considered statistically significant.

### Ethics statement

Animal husbandry and experimental procedures were carried out under approval from the University Committee on Use and Care of Animals, University of Michigan (PRO00005075) and according to Michigan state and US federal regulations. All the mice were housed in specific-pathogen free (SPF) conditions. After weaning, rodent 5001 chow and automatically supplied water were provided ad libitum to mice. Animals were euthanized and analyzed at the specified time points, based on particular study design parameters or defined humane treatment and euthanasia guidelines.

## Supporting Information

S1 Fig Inhibition of *Apc* mutation-induced polyposis in mouse cecum and colon epithelium in the setting of concurrent inactivation of one *Ctnnb1* allele. (A) The intestinal and colon tissues, extending from the terminal ileum through much of the proximal colon, from a control *Apc* ^*fl/fl*^ mouse (lacking Cre). (B and C) Shown are extensive regions of thickened mucosa and polyposis (indicated by the bracket), extending from the terminal ileum (smaller lumen at the left) through much of the proximal colon in a 20-day old *CDX2P-G22Cre Apc* ^*fl/fl*^ mice (B left panel) and in *CDX2P-CreER* ^*T2*^ *Apc* ^*fl/fl*^ mice at 20 days after the third of three daily doses of TAM (C left panel). In contrast, in a 6-month old *CDX2P*-*G22Cre Apc* ^*fl/fl*^ *Ctnnb1* ^*fl/+*^ mice (B right panel) or in *CDX2P-CreER* ^*T2*^ *Apc* ^*fl/fl*^ *Ctnnb1* ^*fl/+*^ mice at 2 or 6 months following TAM induction (C middle and right panels), only occasional isolated adenomas in the cecum were observed (indicated by arrows). Scale bars, 1 cm. (D) Left panels, diagram of the conditionally targeted alleles of *Apc* and *Ctnnb1* (flox allele) and the deletion-mutant alleles of *Apc* and *Ctnnb1* that result from the Cre-mediated recombination of *loxp* sites (floxdel allele). The involved exons are shown as white boxes and the introns are shown as solid lines. Black triangles indicate *loxP* sites. Positions of PCR primers used for the detection of each allele are indicated (P3, P4 and P5 for *Apc* genotyping; RM41, RM42 and YF530 for *Ctnnb1* genotyping). Right panels, Cre-mediated recombination of *Apc* and *Ctnnb1* genes was assessed by PCR using genomic DNA isolated from grossly normal-appearing proximal colon or cecum tissues and rare cecal tumors from three different *CDX2P-CreER* ^*T2*^ *Apc* ^*fl/fl*^ *Ctnnb1* ^*fl/+*^ mice (labeled as obtained from mouse 1, 2, 3) at 6 months following TAM treatment of the mice. Proximal colon or cecal tissue from a *CDX2P-CreER* ^*T2*^ *Apc* ^*fl/fl*^ mouse (labeled as CA), obtained 20 days after TAM induction, or from an *Apc* ^*fl/fl*^ *Ctnnb1* ^*fl/+*^ mouse (lacking the *CDX2P-CreER* ^*T2*^ transgene) (labeled as AB) served as controls. All of the tissues were genotyped for *Apc* and *Ctnnb1*. Flox alleles represent alleles that did not undergo Cre-mediated targeting, whereas the floxdel alleles have undergone Cre-dependent targeting. Due to the PCR primers used to detect the Cre-targeted *Ctnnb1* allele (i.e., foxdel allele), the PCR product for the floxdel allele is larger than that for the flox allele. Tissues were manually dissected. Cecum tumor DNA preparations likely include DNA from some adjacent colon epithelial cells with *Ctnnb1* gene targeting by Cre to generate floxdel alleles. Both epithelial and non-epithelial elements were present in grossly normal-appearing proximal colon and cecum tissue samples from *CDX2P-CreER* ^*T2*^ *Apc* ^*fl/fl*^ *Ctnnb1* ^*fl/+*^ mice. The *Cre* transgene is only expressed in epithelial elements. Both flox and floxdel alleles for *Apc* and *Ctnnb1* are therefore seen in grossly normal-appearing tissue samples from *CDX2P-CreER* ^*T2*^ *Apc* ^*fl/fl*^ *Ctnnb1* ^*fl/+*^ mice; and rare *Apc* flox alleles are seen in proximal colon and cecal tumors due to non-neoplastic elements in the tumors.
(TIF)

S2 Fig Inactivation of one *Ctnnb1* allele inhibits misalignment of the mitotic spindle axis with respect to the planar axis in *Apc*-mutant colon epithelial cells. (A) Proximal colon tissues from wild type (Control, left), *CDX2P-CreER* ^*T2*^ *Apc* ^*fl/fl*^ (middle), or *CDX2P-CreER* ^*T2*^ *Apc* ^*fl/fl*^ *Ctnnb1* ^*fl/+*^ (right) mice, obtained at 27 days after TAM dosing, were co-stained for α-tublin (green), lysozyme (red), and Crb3 (red), and counter-stained with Hoechst 33342 (Blue). Representative low magnification (upper row panels) or high magnification (lower row panels) images are shown. Scale bars, 20 μm for low magnification images, and 10 μm for high magnification images. Double-headed arrows indicate the orientation of mitotic spindles; solid lines indicate the most adjacent apical membrane of the mitotic cell, which was used as a reference to assess the spindle angle. (B) The spindle angles were defined by the orientation of mitotic spindles relative to the most adjacent apical membrane, as indicated by Crb3 staining. Quantification for the tissues is shown and for *CDX2P-CreER* ^*T2*^ *Apc* ^*fl/fl*^ and *CDX2P-CreER* ^*T2*^ *Apc* ^*fl/fl*^ *Ctnnb1* ^*fl/+*^ mice, only the lysozyme-positive crypts, indicating *Apc* gene targeting had occurred, were scored. *P < .01 compared *CDX2P-CreER* ^*T2*^ *Apc* ^*fl/fl*^ mice to *CDX2P-CreER* ^*T2*^ *Apc* ^*fl/fl*^ *Ctnnb1* ^*fl/+*^ mice; n.s., not significant, compared *CDX2P-CreER* ^*T2*^ *Apc* ^*fl/fl*^ *Ctnnb1* ^*fl/+*^ mice to the normal tissues using Chi-Square test (n = 4 and >70 crypts were counted).
(TIF)

S3 Fig Inactivation of one *Ctnnb1* allele in *Apc*-mutant mouse colon epithelium reduces expression of selected Wnt target genes and restricts Sox9 expression to the crypt base region. (A) Proximal colon tissues were co-stained for EphB3 (left, red) and Sox9 (right, green), and counter-stained with Hoechst 33342 (Blue). The tissues from a *CDX2P-CreER* ^*T2*^ *Apc* ^*fl/fl*^ *Ctnnb1* ^*fl/+*^ mouse (middle) and a *CDX2P-CreER* ^*T2*^ *Apc* ^*fl/fl*^ mouse (bottom) were analyzed 20 days after the third of three daily doses of TAM. The normal colon mucosa from a *Cre*-negative mouse was used as a control (Cntrl, top). Scale bars, 20 μm. (B) Detection of *Axin2* locus-regulated LacZ reporter (*Axin2-LacZ*) gene expression in mouse proximal colon tissues, as assessed by X-gal staining with H&E counter-staining. Tissues from the *CDX2P-CreER* ^*T2*^ *Apc* ^*fl/fl*^ *Ctnnb1* ^*fl/+*^ mouse (middle) and the *CDX2P-CreER* ^*T2*^ *Apc* ^*fl/fl*^ mouse (bottom) with the reporter gene *Axin2-LacZ* were analyzed 20 days following two daily doses of TAM. The normal colon mucosa from a wild-type mouse (Cntrl) and a *Cre* negative reporter mouse (Axin2-LacZ) were used as controls (top). Scale bars, 50 μm.
(TIF)

S4 Fig Inactivation of one *Ctnnb1* allele in *Apc*-mutant mouse colon epithelium inhibits *Apc* mutation-induced increases in the expression of Wnt/β-catenin/TCF pathway target genes and genes encoding stem cell markers. (A) Effects of *Ctnnb1* inactivation on *Apc* mutation-induced expression of selected Wnt target genes, *Axin2*, *Nkd1*, *Ccnd1*, and *Irs1*. Gene expression was assessed by qRT-PCR in mouse proximal colon tissues obtained from Cre negative control mice (cntrl), *CDX2P-CreER* ^*T2*^ *Apc* ^*fl/fl*^ *Ctnnb1* ^*fl/+*^ mice (*Apc* ^*fl/fl*^ *Ctnnb1* ^*fl/+*^) and *CDX2P-CreER* ^*T2*^ *Apc* ^*fl/fl*^ mice (*Apc* ^*fl/fl*^) at 20 days after TAM dosing to activate Cre to target alleles. Gene expression was normalized to *β-actin* expression. (B) Effects of *Ctnnb1* inactivation on *Apc* mutation-induced expression of selected candidate stem cell markers, *Lgr5*, *CD44*, *Msi1* and *Hopx* (*Lgr5* and *CD44* are also Wn/β-catenin/TCF pathway target genes). RNA preparations used for the work in panel A were studied by qRT-PCR and normalized to *β-actin* expression. Two *asterisks* denote *P* < 0.01 and one *asterisk* denotes *P* < 0.05 in Student's *t* test, and *error bars* denote S.D. (n = 3 for each group).
(TIF)

S5 Fig Inhibition of *APC* gene and protein expression by a DOX-regulated shRNA approach in HCECs. HCECs stably transduced with a lentiviral vector (ShRNA V1) expressing an *APC* shRNA or a non-silencing scramble shRNA (Scrmbl) were further transduced with lentiviral vectors (ShRNA V2) driving expression of two different shRNAs targeting *CTNNB1* (*CTNNB1*-1 and *CTNNB1*-2) or a non-silencing scramble shRNA (Scrmbl). RNA and protein were collected from the HCECs at 3 days in the presence of doxycycline (DOX, “+”) at 2 μg/ml or simply in a solvent control lacking DOX (“-“). (A) Inhibition of *APC* gene expression by the DOX-inducible *APC* shRNA construct was demonstrated by gene expression analysis by qRT-PCR, with normalization to *U6* expression (n = 3; **P < 0.01). (B) Inhibition of APC protein expression by the DOX-inducible *APC* shRNA construct was demonstrated by Western blot analysis with two different antibodies against APC (Ab-5 and C-20). β-actin was used as a loading and transfer control.
(TIF)

S6 Fig The active pool of β-catenin protein is highly sensitive to changes in β-catenin transcript levels in HCEC cells. (A) HCECs stably transduced with a lentiviral vector (ShRNA V1) expressing an *APC* shRNA or a non-silencing scramble shRNA (Scrmbl) were further transduced with lentiviral vectors (ShRNA V2) driving expression of two different shRNAs targeting *CTNNB1* (*CTNNB1*-1 and *CTNNB1*-2) or a non-silencing scramble shRNA (Scrmbl). Proteins were collected from the HCECs at 3 days in the presence of doxycycline (DOX, “+”) at 2 μg/ml or simply in a solvent control lacking DOX (“-“). *CTNNB1* shRNA-mediated changes in the expression of the total (right) and active (un- or hypophosphorylated, left) pools of β-catenin in the HCECs were assessed by Western blot analysis (Fig 7A) and the density of Western blotting bands was quantified using AlphaImager HP system (from ProteinSimple). The protein levels of total and active β-catenin were normalized to β-actin level, and the expression from HCEC/Scrmbl in the presence of DOX was set as 1. (B) Similar Western blot analysis was performed as described in (A) except that the HCECs were treated with doxycycline (DOX, “+”) at 1 μg/ml or 2 μg/ml or simply in a solvent control lacking DOX (“-“). The protein levels of total and active β-catenin were shown (top) and the density of Western blotting bands was quantified and shown at the bottom panels. β-actin was used as a loading and transfer control.
(TIF)

S7 Fig Immunohistochemical studies in HCECs demonstrate that the nuclear and cytoplasmic pools of β-catenin protein induced by *APC* inhibition are markedly affected by reduction of *Ctnnb1* transcript levels. HCECs stably transduced with a lentiviral vector (ShRNA V1) expressing an *APC* shRNA or a non-silencing scramble shRNA (Scrmbl) were further transduced with lentiviral vectors (ShRNA V2) driving expression of two different shRNAs targeting *CTNNB1* (*CTNNB1*-1 and *CTNNB1*-2) or a non-silencing scramble shRNA (Scrmbl). These cells were grown on chamber slides and were induced for 3 days for shRNA expression by addition of DOX at 2 μg/ml. Cells were then subjected to Immunohistochemical staining for β-catenin. Scale bar, 20 μm.
(TIF)

S8 Fig The active (non-phosphorylated) pool of β-catenin that functions in canonical Wnt signaling through interactions with TCFs is more sensitive to shRNA-mediated inhibition of *CTNNB1* transcripts than the total pool of β-catenin in human CRC cell lines with mutational defects in Wnt signaling. Western blot analysis of protein levels of non-phosphorylated, active β-catenin and total β-catenin in human colon cancer cell lines, DLD1 (A), HCT116 (B) and SW480 (C) (with APC inactivation for DLD1 and SW480; β-catenin gain of function for HCT116), stably expressing two different doxycycline-inducible shRNAs targeting *CTNNB1* (*CTNNB1*-1 and *CTNNB1*-2) or a non-silencing scramble shRNA (Scrmbl). β-catenin protein levels were determined after 3-day exposure of the cells to DOX (“+”) at 2 μg/ml or a solvent control (“-“). β-actin protein levels served as a loading and transfer control. The density of Western blotting bands for each cell lines was quantified using AlphaImager HP system (from ProteinSimple). The protein levels of active and total β-catenin were normalized to β-actin level, and the expression from cells expressing scramble shRNA in the presence of DOX was set as 1.
(TIF)

S9 Fig Inhibition of *CTNNB1* expression by RNA interference reduces expression of Wnt/β-catenin/TCF pathway target genes in human colon cancer cells harboring mutations in the Wnt pathway factors APC (DLD1 cells) or β-catenin (HCT116 cells). DLD1 and HCT116 cells stably transduced with two different doxycycline-inducible shRNAs targeting *CTNNB1* (*CTNNB1*-1 and *CTNNB1*-2) or a non-silencing scramble shRNA (Scrmbl) were treated for 7 days with DOX (“+ DOX”) at 2 μg/ml or a solvent control (“- DOX”). Analysis of the β-catenin dosage effects on gene expression are shown: *CTNNB1* (A); and Wnt target genes—*AXIN2* (B), *BMP4* (C), *NKD1* (D), *LGR5* (E) and *CD44* (F). Gene expression was assessed by qRT-PCR and normalized to *HPRT* expression. *Error bars* denote S.D. **P < 0.01 and *P < 0.05.
(TIF)

S1 Table Primer sequence for qRT-PCR. (DOCX)
