# Supplementary figures and images for: Tissue-Specific Effects of Reduced β-catenin Expression on Adenomatous Polyposis Coli Mutation-Instigated Tumorigenesis in Mouse Colon and Ovarian Epithelium
Source: PLoS Genet. 2015 Nov 3;11(11):e1005638. doi: 10.1371/journal.pgen.1005638 (PMC4631511; doi:10.1371/journal.pgen.1005638)

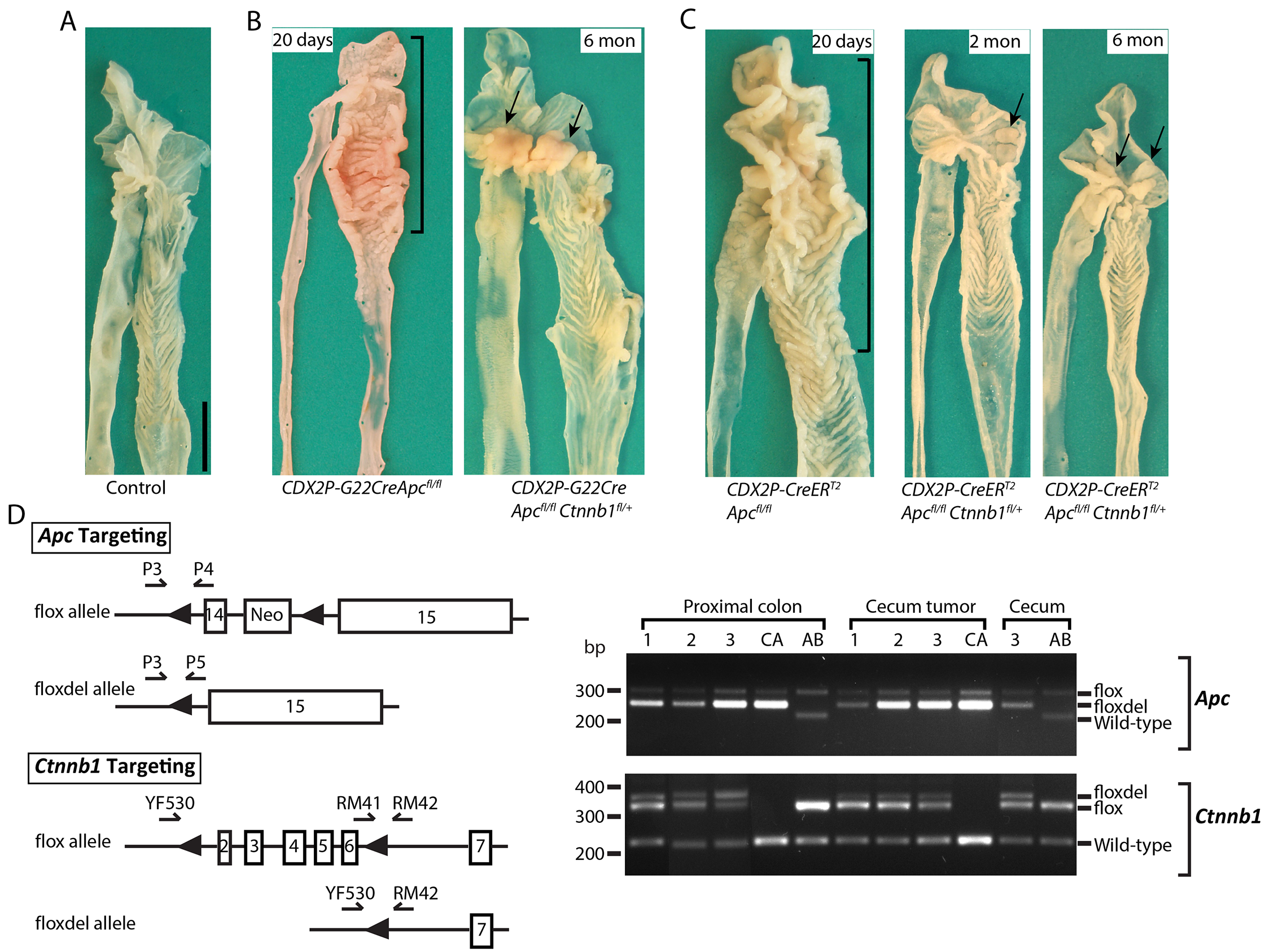

Supplement: S1 Fig — (A) The intestinal and colon tissues, extending from the terminal ileum through much of the proximal colon, from a control Apc fl/fl mouse (lacking Cre). (B and C) Shown are extensive regions of thickened mucosa and polyposis (indicated by the bracket), extending from the terminal ileum (smaller lumen at the left) through much of the proximal colon in a 20-day old CDX2P-G22Cre Apc fl/fl mice (B left panel) and in CDX2P-CreER T2 Apc fl/fl mice at 20 days after the third of three daily doses of TAM (C left panel). In contrast, in a 6-month old CDX2P-G22Cre Apc fl/fl Ctnnb1 fl/+ mice (B right panel) or in CDX2P-CreER T2 Apc fl/fl Ctnnb1 fl/+ mice at 2 or 6 months following TAM induction (C middle and right panels), only occasional isolated adenomas in the cecum were observed (indicated by arrows). Scale bars, 1 cm. (D) Left panels, diagram of the conditionally targeted alleles of Apc and Ctnnb1 (flox allele) and the deletion-mutant alleles of Apc and Ctnnb1 that result from the Cre-mediated recombination of loxp sites (floxdel allele). The involved exons are shown as white boxes and the introns are shown as solid lines. Black triangles indicate loxP sites. Positions of PCR primers used for the detection of each allele are indicated (P3, P4 and P5 for Apc genotyping; RM41, RM42 and YF530 for Ctnnb1 genotyping). Right panels, Cre-mediated recombination of Apc and Ctnnb1 genes was assessed by PCR using genomic DNA isolated from grossly normal-appearing proximal colon or cecum tissues and rare cecal tumors from three different CDX2P-CreER T2 Apc fl/fl Ctnnb1 fl/+ mice (labeled as obtained from mouse 1, 2, 3) at 6 months following TAM treatment of the mice. Proximal colon or cecal tissue from a CDX2P-CreER T2 Apc fl/fl mouse (labeled as CA), obtained 20 days after TAM induction, or from an Apc fl/fl Ctnnb1 fl/+ mouse (lacking the CDX2P-CreER T2 transgene) (labeled as AB) served as controls. All of the tissues were genotyped for Apc and Ctnnb1. Flox alleles represent alleles [file pgen.1005638.s001.tif]

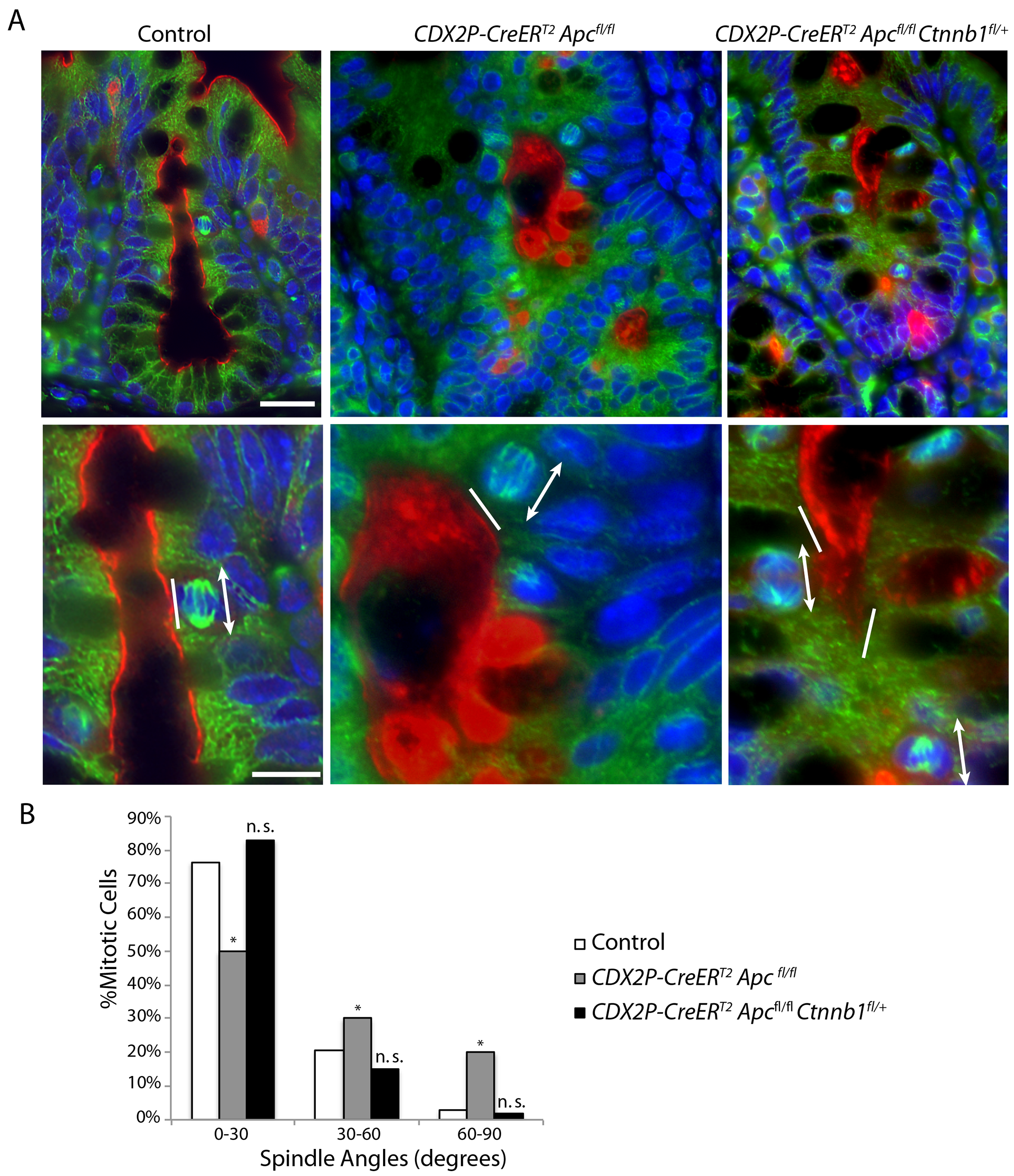

Supplement: S2 Fig — (A) Proximal colon tissues from wild type (Control, left), CDX2P-CreER T2 Apc fl/fl (middle), or CDX2P-CreER T2 Apc fl/fl Ctnnb1 fl/+ (right) mice, obtained at 27 days after TAM dosing, were co-stained for α-tublin (green), lysozyme (red), and Crb3 (red), and counter-stained with Hoechst 33342 (Blue). Representative low magnification (upper row panels) or high magnification (lower row panels) images are shown. Scale bars, 20 μm for low magnification images, and 10 μm for high magnification images. Double-headed arrows indicate the orientation of mitotic spindles; solid lines indicate the most adjacent apical membrane of the mitotic cell, which was used as a reference to assess the spindle angle. (B) The spindle angles were defined by the orientation of mitotic spindles relative to the most adjacent apical membrane, as indicated by Crb3 staining. Quantification for the tissues is shown and for CDX2P-CreER T2 Apc fl/fl and CDX2P-CreER T2 Apc fl/fl Ctnnb1 fl/+ mice, only the lysozyme-positive crypts, indicating Apc gene targeting had occurred, were scored. *P < .01 compared CDX2P-CreER T2 Apc fl/fl mice to CDX2P-CreER T2 Apc fl/fl Ctnnb1 fl/+ mice; n.s., not significant, compared CDX2P-CreER T2 Apc fl/fl Ctnnb1 fl/+ mice to the normal tissues using Chi-Square test (n = 4 and >70 crypts were counted). (TIF) [file pgen.1005638.s002.tif]

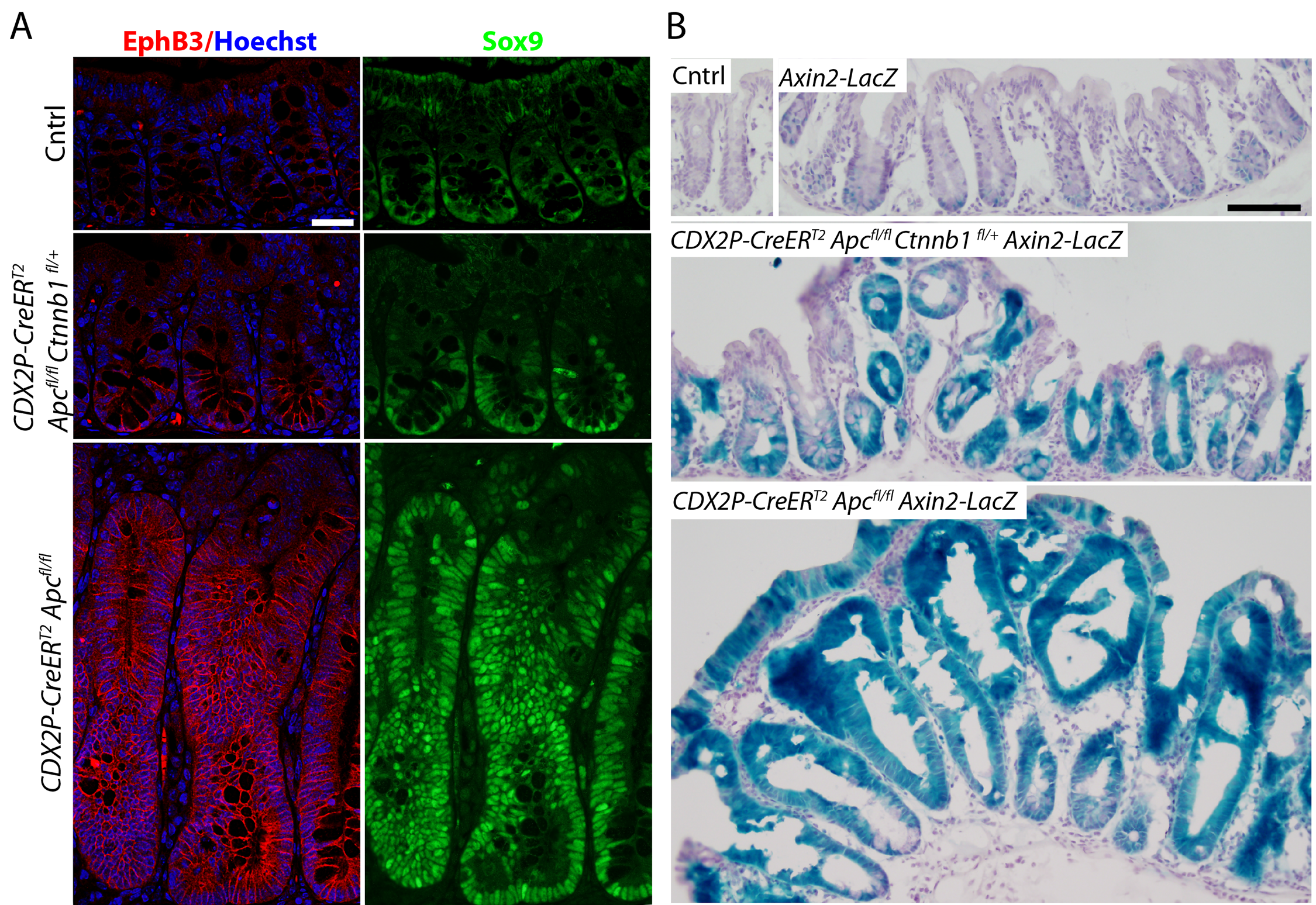

Supplement: S3 Fig — (A) Proximal colon tissues were co-stained for EphB3 (left, red) and Sox9 (right, green), and counter-stained with Hoechst 33342 (Blue). The tissues from a CDX2P-CreER T2 Apc fl/fl Ctnnb1 fl/+ mouse (middle) and a CDX2P-CreER T2 Apc fl/fl mouse (bottom) were analyzed 20 days after the third of three daily doses of TAM. The normal colon mucosa from a Cre-negative mouse was used as a control (Cntrl, top). Scale bars, 20 μm. (B) Detection of Axin2 locus-regulated LacZ reporter (Axin2-LacZ) gene expression in mouse proximal colon tissues, as assessed by X-gal staining with H&E counter-staining. Tissues from the CDX2P-CreER T2 Apc fl/fl Ctnnb1 fl/+ mouse (middle) and the CDX2P-CreER T2 Apc fl/fl mouse (bottom) with the reporter gene Axin2-LacZ were analyzed 20 days following two daily doses of TAM. The normal colon mucosa from a wild-type mouse (Cntrl) and a Cre negative reporter mouse (Axin2-LacZ) were used as controls (top). Scale bars, 50 μm. (TIF) [file pgen.1005638.s003.tif]

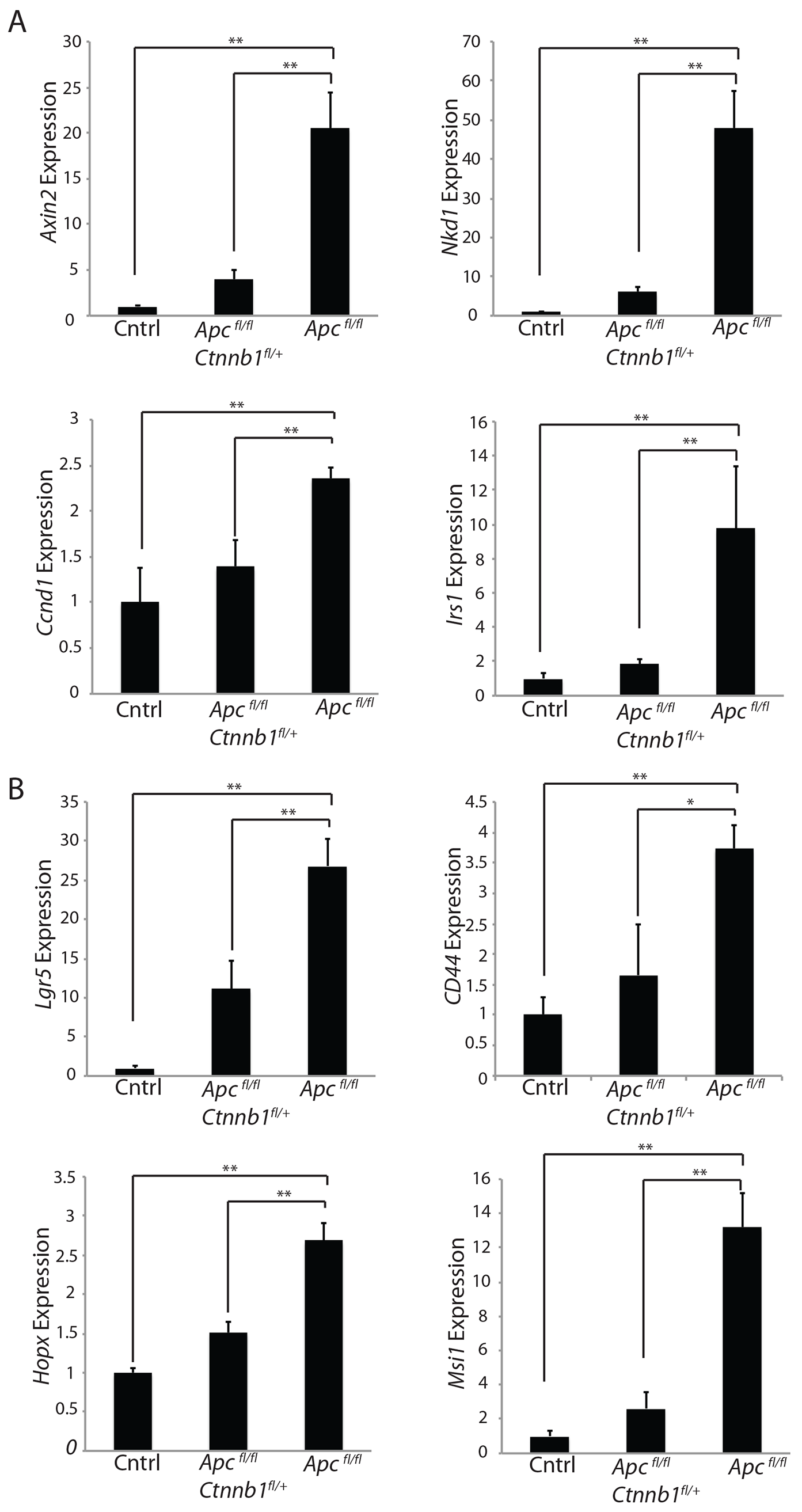

Supplement: S4 Fig — (A) Effects of Ctnnb1 inactivation on Apc mutation-induced expression of selected Wnt target genes, Axin2, Nkd1, Ccnd1, and Irs1. Gene expression was assessed by qRT-PCR in mouse proximal colon tissues obtained from Cre negative control mice (cntrl), CDX2P-CreER T2 Apc fl/fl Ctnnb1 fl/+ mice (Apc fl/fl Ctnnb1 fl/+) and CDX2P-CreER T2 Apc fl/fl mice (Apc fl/fl) at 20 days after TAM dosing to activate Cre to target alleles. Gene expression was normalized to β-actin expression. (B) Effects of Ctnnb1 inactivation on Apc mutation-induced expression of selected candidate stem cell markers, Lgr5, CD44, Msi1 and Hopx (Lgr5 and CD44 are also Wn/β-catenin/TCF pathway target genes). RNA preparations used for the work in panel A were studied by qRT-PCR and normalized to β-actin expression. Two asterisks denote P < 0.01 and one asterisk denotes P < 0.05 in Student's t test, and error bars denote S.D. (n = 3 for each group). (TIF) [file pgen.1005638.s004.tif]

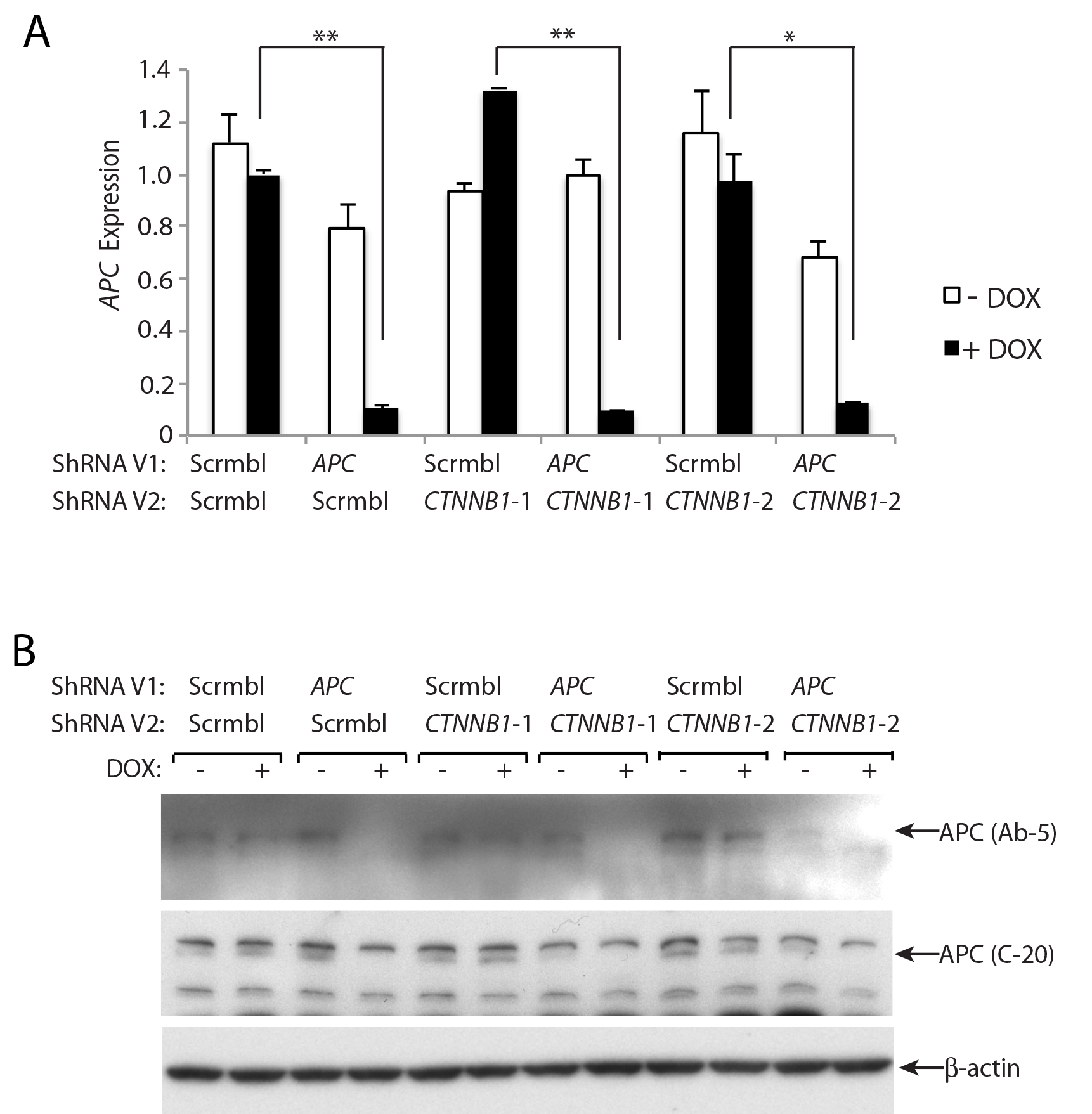

Supplement: S5 Fig — HCECs stably transduced with a lentiviral vector (ShRNA V1) expressing an APC shRNA or a non-silencing scramble shRNA (Scrmbl) were further transduced with lentiviral vectors (ShRNA V2) driving expression of two different shRNAs targeting CTNNB1 (CTNNB1-1 and CTNNB1-2) or a non-silencing scramble shRNA (Scrmbl). RNA and protein were collected from the HCECs at 3 days in the presence of doxycycline (DOX, “+”) at 2 μg/ml or simply in a solvent control lacking DOX (“-“). (A) Inhibition of APC gene expression by the DOX-inducible APC shRNA construct was demonstrated by gene expression analysis by qRT-PCR, with normalization to U6 expression (n = 3; **P < 0.01). (B) Inhibition of APC protein expression by the DOX-inducible APC shRNA construct was demonstrated by Western blot analysis with two different antibodies against APC (Ab-5 and C-20). β-actin was used as a loading and transfer control. (TIF) [file pgen.1005638.s005.tif]

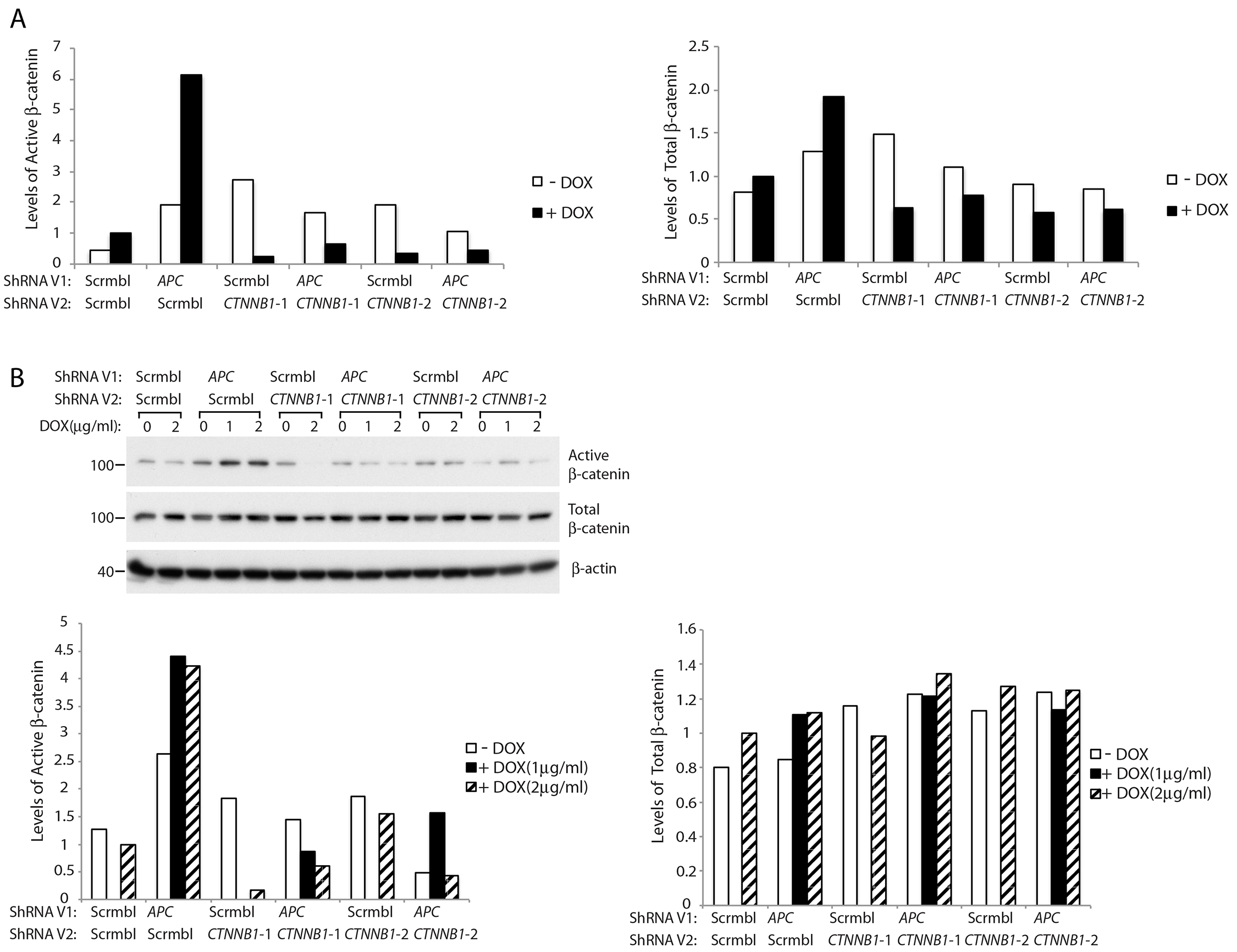

Supplement: S6 Fig — (A) HCECs stably transduced with a lentiviral vector (ShRNA V1) expressing an APC shRNA or a non-silencing scramble shRNA (Scrmbl) were further transduced with lentiviral vectors (ShRNA V2) driving expression of two different shRNAs targeting CTNNB1 (CTNNB1-1 and CTNNB1-2) or a non-silencing scramble shRNA (Scrmbl). Proteins were collected from the HCECs at 3 days in the presence of doxycycline (DOX, “+”) at 2 μg/ml or simply in a solvent control lacking DOX (“-“). CTNNB1 shRNA-mediated changes in the expression of the total (right) and active (un- or hypophosphorylated, left) pools of β-catenin in the HCECs were assessed by Western blot analysis (Fig 7A) and the density of Western blotting bands was quantified using AlphaImager HP system (from ProteinSimple). The protein levels of total and active β-catenin were normalized to β-actin level, and the expression from HCEC/Scrmbl in the presence of DOX was set as 1. (B) Similar Western blot analysis was performed as described in (A) except that the HCECs were treated with doxycycline (DOX, “+”) at 1 μg/ml or 2 μg/ml or simply in a solvent control lacking DOX (“-“). The protein levels of total and active β-catenin were shown (top) and the density of Western blotting bands was quantified and shown at the bottom panels. β-actin was used as a loading and transfer control. (TIF) [file pgen.1005638.s006.tif]

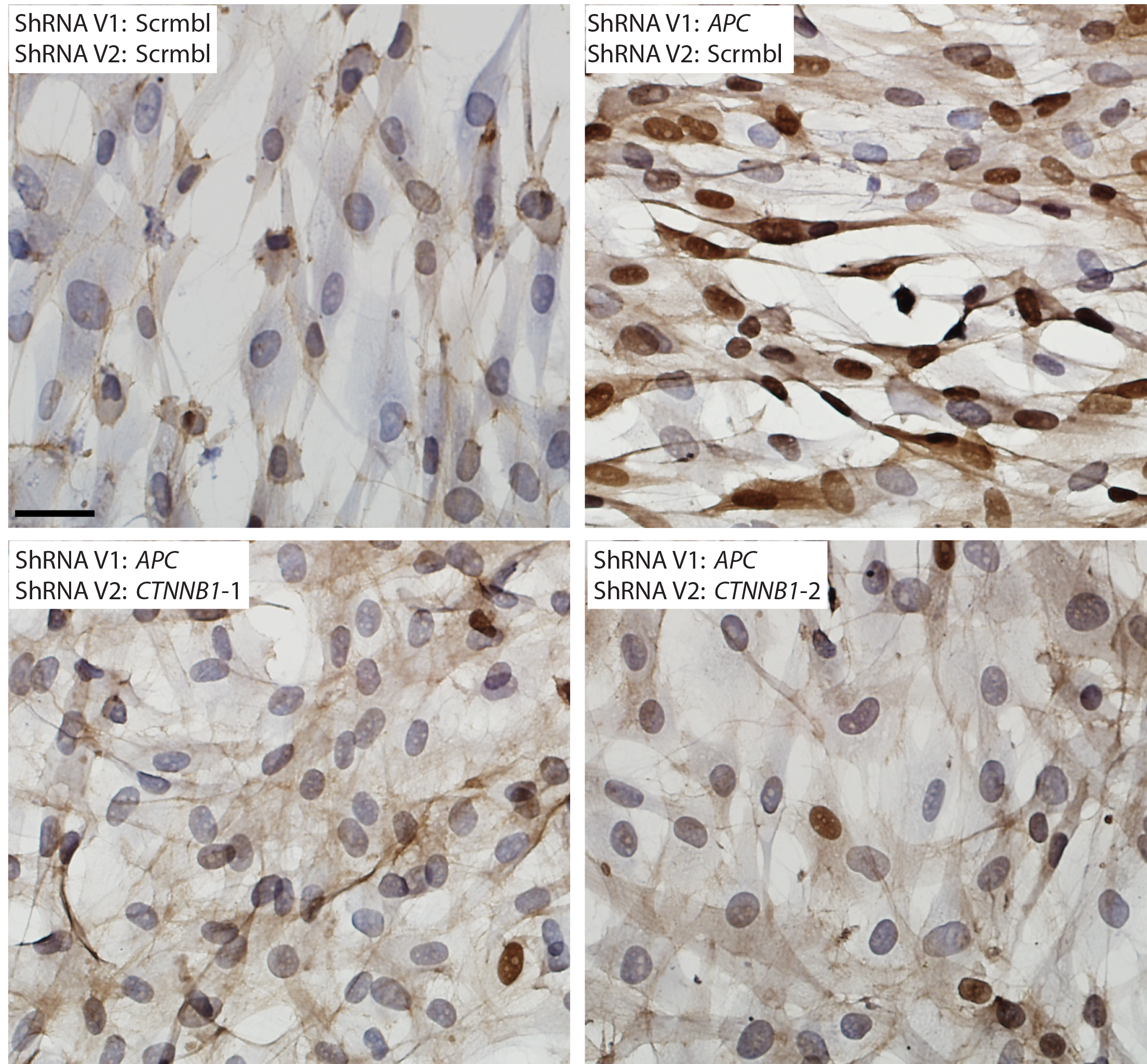

Supplement: S7 Fig — HCECs stably transduced with a lentiviral vector (ShRNA V1) expressing an APC shRNA or a non-silencing scramble shRNA (Scrmbl) were further transduced with lentiviral vectors (ShRNA V2) driving expression of two different shRNAs targeting CTNNB1 (CTNNB1-1 and CTNNB1-2) or a non-silencing scramble shRNA (Scrmbl). These cells were grown on chamber slides and were induced for 3 days for shRNA expression by addition of DOX at 2 μg/ml. Cells were then subjected to Immunohistochemical staining for β-catenin. Scale bar, 20 μm. (TIF) [file pgen.1005638.s007.tif]

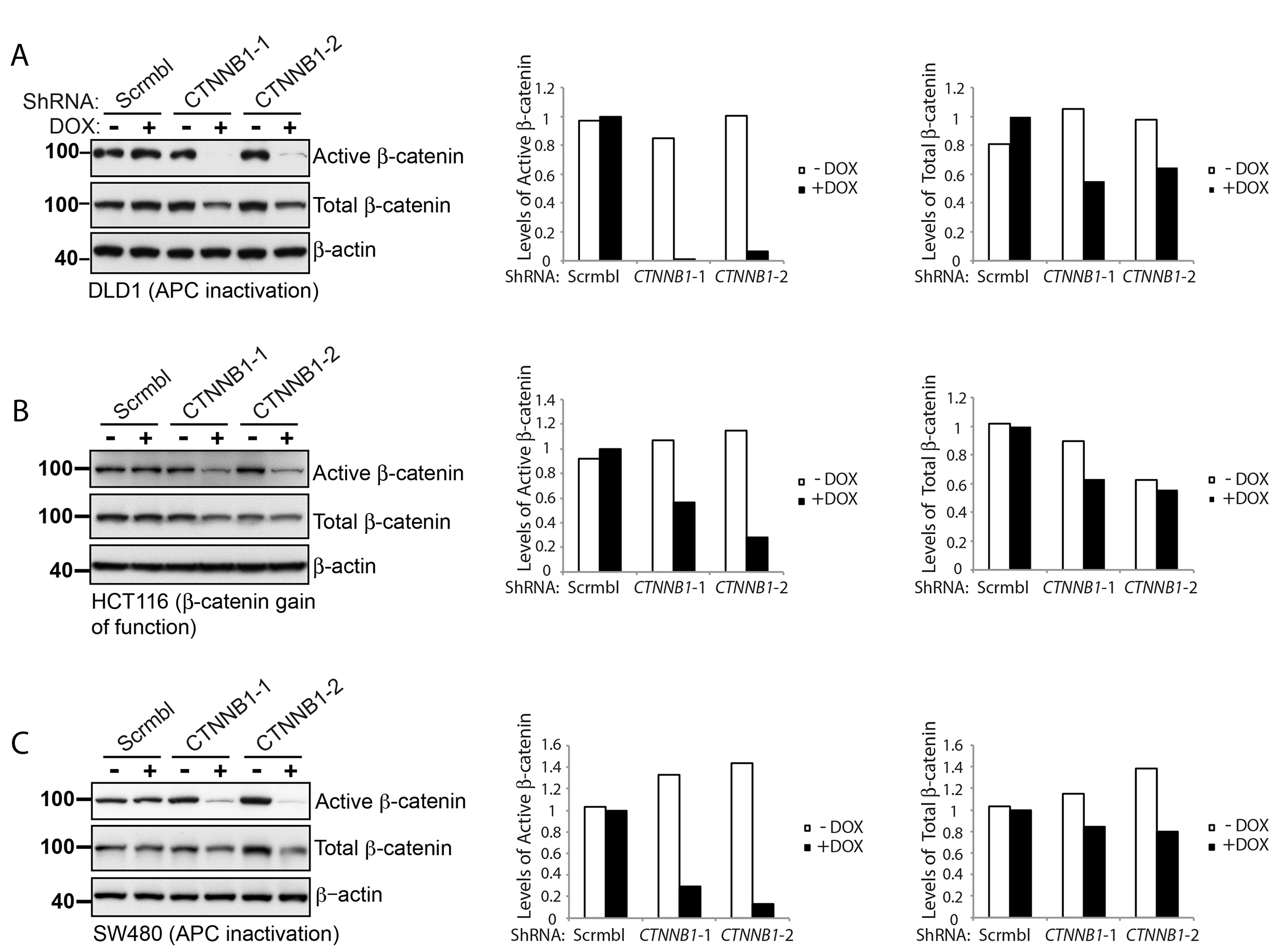

Supplement: S8 Fig — Western blot analysis of protein levels of non-phosphorylated, active β-catenin and total β-catenin in human colon cancer cell lines, DLD1 (A), HCT116 (B) and SW480 (C) (with APC inactivation for DLD1 and SW480; β-catenin gain of function for HCT116), stably expressing two different doxycycline-inducible shRNAs targeting CTNNB1 (CTNNB1-1 and CTNNB1-2) or a non-silencing scramble shRNA (Scrmbl). β-catenin protein levels were determined after 3-day exposure of the cells to DOX (“+”) at 2 μg/ml or a solvent control (“-“). β-actin protein levels served as a loading and transfer control. The density of Western blotting bands for each cell lines was quantified using AlphaImager HP system (from ProteinSimple). The protein levels of active and total β-catenin were normalized to β-actin level, and the expression from cells expressing scramble shRNA in the presence of DOX was set as 1. (TIF) [file pgen.1005638.s008.tif]

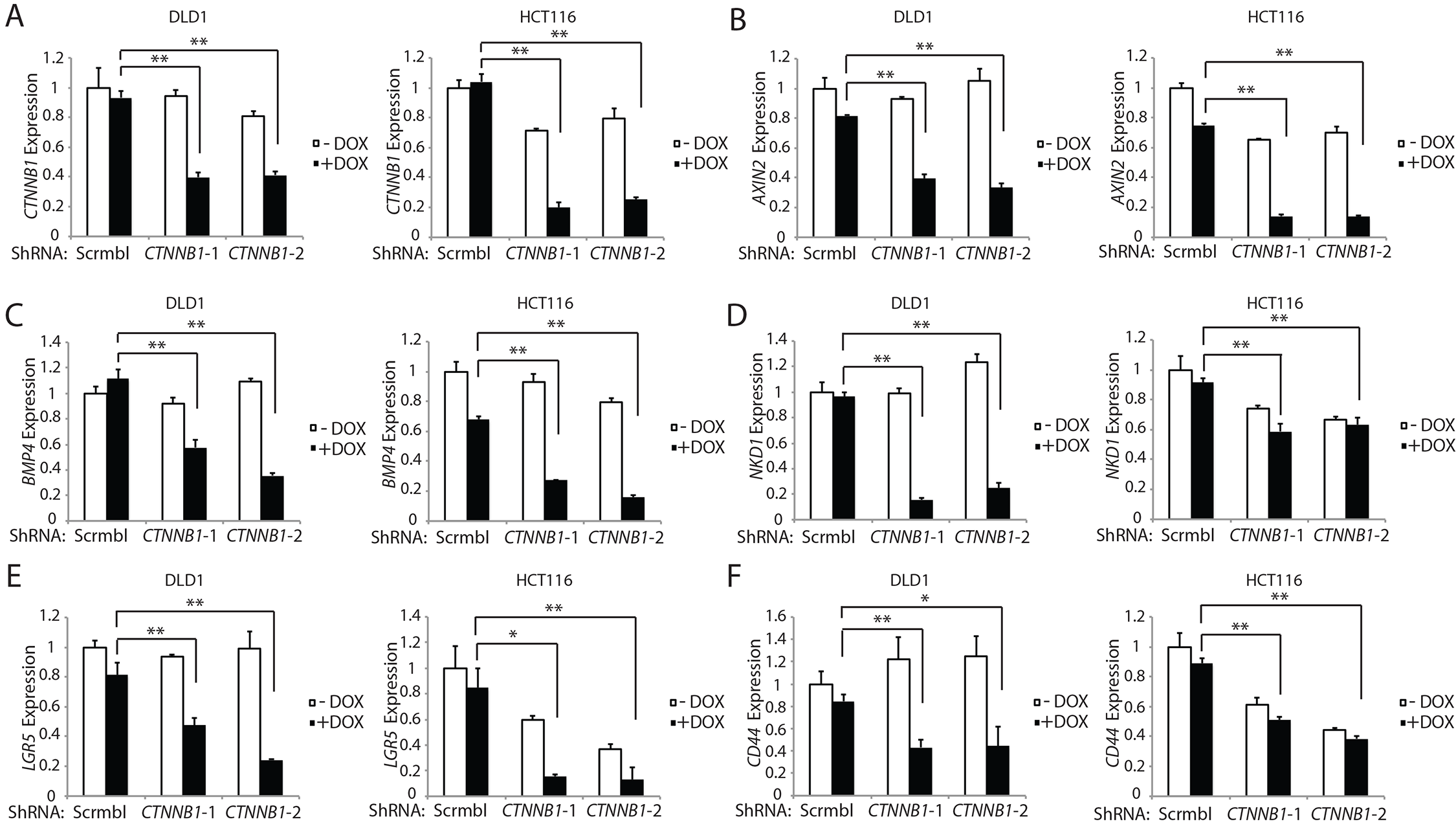

Supplement: S9 Fig — DLD1 and HCT116 cells stably transduced with two different doxycycline-inducible shRNAs targeting CTNNB1 (CTNNB1-1 and CTNNB1-2) or a non-silencing scramble shRNA (Scrmbl) were treated for 7 days with DOX (“+ DOX”) at 2 μg/ml or a solvent control (“- DOX”). Analysis of the β-catenin dosage effects on gene expression are shown: CTNNB1 (A); and Wnt target genes—AXIN2 (B), BMP4 (C), NKD1 (D), LGR5 (E) and CD44 (F). Gene expression was assessed by qRT-PCR and normalized to HPRT expression. Error bars denote S.D. **P < 0.01 and *P < 0.05. (TIF) [file pgen.1005638.s009.tif]
