# Supplementary material for: Tissue-Specific Effects of Reduced β-catenin Expression on Adenomatous Polyposis Coli Mutation-Instigated Tumorigenesis in Mouse Colon and Ovarian Epithelium
Source: PLoS Genet. 2015 Nov 3;11(11):e1005638. doi: 10.1371/journal.pgen.1005638 (PMC4631511; doi:10.1371/journal.pgen.1005638)
Supplement: S1 Table — (DOCX) [file pgen.1005638.s010.docx]

**S1 Table. Primer Sequence for qRT-PCR**

| **Primers for Human Genes** | |
| --- | --- |
| Primer Name | Sequence |
| NKD1-F | 5’-catgcacgacatgaaaatcc-3’ |
| NKD1-R | 5’-ttgcctggatcttggaaaac-3’ |
| BMP4-F | 5’-tgagcctttccagcaagttt-3’ |
| BMP4-R | 5’-cttccccgtctcaggtatca-3’ |
| CD44-F | 5’-agcaaccaagaggcaagaaacct-3’ |
| CD44-R | 5’-gcccgtggtgtggttgaaatg-3’ |
| LGR5-F | 5’-TGCTCTTCACCAACTGCATC-3’ |
| LGR5-R | 5’-CTCAGGCTCACCAGATCCTC-3’ |
| IRS1-F | 5’-CCAGAAGCAGCCAGAGGA-3’ |
| IRS1-R | 5’-CCCATGAGTTAGAAGAGGATTTG-3’ |
| AXIN2-F | 5’-CACACCCTTCTCCAATCCAA-3’ |
| AXIN2-R | 5’-CACAGAAAAAGTAAGTGACAACCA –3’ |
| CTNNB1-F | 5’-GTCTGAGGACAAGCCACAAG-3’ |
| CTNNB1-R | 5’-GGGCACCAATATCAAGTCCA-3’ |
| HPRT1-F | 5’-TGACACTGGCAAAACAATGCA-3’ |
| HPRT1-R | 5’-GGTCCTTTTCACCAGCAAGCT-3’ |
| U6-F | 5’-GTGCTCGCTTCGGCAGCACATAT-3’ |
| U6-R | 5’-AAAAATATGGAACGCTTCACGAA-3’ |
| MYC- F | 5’-TTTCGGGTAGTGGAAAACCA-3’ |
| MYC-R | 5’-CAGCAGCTCGAATTTCTTCC-3’ |
| **Primers for Mouse Genes** | |
| Primer Name | Sequence |
| Ccnd1-F | 5’-CAGAGGCGGATGAGAACAA-3’ |
| Ccnd1-R | 5’-AGGGTGGGTTGGAAATGAA-3’ |
| Axin2-F | 5’-GAGGATGCTGAAGGCTCAAA-3’ |
| Axin2-R | 5’-GCAGGCAAATTCGTCACTC-3’ |
| Irs1-F | 5’-GACGCTCCAGTGAGGATTT-3’ |
| Irs1-R | 5’-AGGTCCTGGTTGTGAATTGTG-3’ |
| Msi1-F | 5’-CCCCTCCAGGTTCCACTC-3’ |
| Msi1-R | 5’-GTGAAGGCTGTGGCAATCAA-3’ |
| Lgr5-F | 5’-GGAATAAAGACGACGGCAAC-3’ |
| Lgr5-R | 5’-GGATCAGCCAGCTACCAAA-3’ |
| Cd44-F | 5’-GGCTCATCATCTTGGCATCT-3’ |
| Cd44-R | 5’-CACTGGGTTTCCTGTCTTCC-3’ |
| Nkd1-F | 5’-CCGAGCAGACACCAAACC-3’ |
| Nkd1-R | 5’-TCCTCTCAATGTTCTCATCCA-3’ |
| Hopx-F | 5’-CACCACGCTGTGCCTCAT-3’ |
| Hopx-R | 5’-GGCTCCCTAGTCCGTAACA-3’ |
| β-actin-F | 5’-agccatgtacgtagccatcc-3’ |
| β-actin-R | 5’-ctctcagctgtggtggtgaa-3’ |
| Ctnnb1-F | 5’-AATGGCTTGGAATGAGACTG-3’ |
| Ctnnb1-R | 5’-ACCAGAGTGAAAAGAACGGTAG-3’ |
| Epcam-F | 5’-AACACAAGACGACGTGGACA-3’ |
| Epcam-R | 5’-GCTCTCCGTTCACTCTCAGG-3’ |
| Cdh1-F | 5’- GTTGTGCTCAAGCCTTCAC-3’ |
| Cdh1-R | 5’- GTCAACACCTACAACGCTGCC-3’ |
